# Development and application of whole genome SNP markers in mango

**DOI:** 10.1186/s12864-026-13182-z

**Published:** 2026-07-18

**Authors:** Feng Liu, Chunyuan Wu, Jingping Liu, Quansheng Yao, Ru-lin Zhan

**Affiliations:** 1 South Subtropical Crops Research Institute, Chinese Academy of Tropical Agricultural Sciences, Key Laboratory of Postharvest Physiology and Technology of Tropical Horticultural Products of Hainan Province, Zhanjiang, Guangdong 524091 China; 2 State Key Laboratory of Tropical Crop Breeding, Sanya, 572024 China

**Keywords:** *Mangifera indica*, Single nucleotide polymorphisms, Germplasm resources, DNA fingerprinting, Hybrid authentication, SNP marker

## Abstract

**Background:**

Mango (*Mangifera indica* L.) is a globally significant fruit over the world. The precise identification of germplasm resources is of great significance for the breeding of new mango cultivars. Traditional methods based on phenotypic characterization are time-consuming, labor-intensive, and often lack accuracy in distinguishing and identifying accessions. In contrast, DNA fingerprinting utilizing single nucleotide polymorphism (SNP) markers offers an efficient and accurate alternative for germplasm identification and variety protection. However, applications of such SNP-based systems remain relatively limited in mango research.

**Results:**

In this study, 438,322 SNP sites of SNP—InDel information were obtained by GATK software filtering. After filtered by minimum allele frequency (MAF) > 0.4; mismatch rate < 0.25; heterozygosity rate < 0.5; no other mutations within 70 bp on either side of the SNP site, 272 high-quality SNP sites was identified. Then 85 novel PARMS-SNP markers were developed. Based on the 85 novel PARMS-SNP markers, 145 mango germplasm accessions were grouped to three major categories. Based on the polymorphism results, 16 SNP markers were selected as the core markers to construct the DNA fingerprints of 145 mango germplasms. The results showed that 145 mango germplasms had unique genotyping and strong identification ability, indicating that there was no misidentification in the germplasm. At the same time, it provided a basis for the accurate identification of mango germplasm resources and the breeding of new varieties. In addition, 85 SNP markers were screened and 3 specific SNP markers of “Keitt” (KT) × “Renong No.1” (RN) were mined, which could effectively distinguish parents and control materials. The most important thing was to realize the rapid identification of mango parent–child relationship.

**Conclusions:**

To the best of our knowledge, this represents the first attempt to establish SNP markers in genomic wide. The objective is to conduct genetic diversity analysis and construct DNA fingerprint of mango germplasm resources, thereby providing a foundation for germplasm utilization and molecular marker-assisted breeding.

**Supplementary Information:**

The online version contains supplementary material available at https://doi.org/10.1186/s12864-026-13182-z.

## Background

The mango genus (*Mangifera* L.) belongs to the Anacardiaceae family. It was first mentioned in Indian literature as early as 2000 [1] BCE and is one of the world's most important tropical fruit trees, possessing significant nutritional, economic, and medicinal value. There are 41 confirmed species within the mango genus, with approximately 15 producing edible fruit. The primary species cultivated worldwide is the mango (*M.indica* L), with over 1,000 varieties currently in existence globally [2, 3].

However, genetic admixture and confusion in taxonomic relationships have emerged due to the complex process of mango domestication from its origins and the cross-regional introduction of varieties. This has created significant challenges for mango variety management and poses considerable difficulties in classifying germplasm resources. Therefore, establishing a rapid, accurate, reliable, and efficient method for identifying mango germplasm resources is crucial for better protecting existing mango variety rights and authenticating new varieties. This approach is vital for ensuring the authenticity of mango germplasm and safeguarding variety protection.

The development of molecular markers has provided essential tools for the efficient identification and genetic improvement of germplasm resources. Unlike traditional morphological evaluation methods, DNA molecular markers offer advantages such as high polymorphism, strong stability, and rich genetic information. They have been widely applied in germplasm identification, phylogenetic analysis, genetic diversity research, DNA fingerprint profiling, linkage map development, and molecular-assisted breeding [4]. The molecular markers reported in mango research primarily include RAPD [5], ISSR [6],SSR [7], and AFLP [8]. Although these marker technologies have to some extent facilitated the advancement of mango germplasm resource research, they still face limitations such as low detection throughput, poor reproducibility, and low genotyping efficiency, making it challenging to meet the demands of the rapidly developing mango industry.

Single Nucleotide Polymorphism (SNP) markers were first proposed by American scholars LandEre and Eberle in 1996 as a type of molecular marker [9]. They are currently the most common type of genomic variation, widely distributed across biological genomes. SNPs are characterized by stable mutations, high information content, and suitability for automated high-throughput detection, making them significant in biological breeding and medical disease research [10]. SNP molecular markers have the advantages of rich loci, wide distribution, high degree of automation, good stability and strong representativeness [11, 12]. Therefore, it has become one of the most promising molecular markers and has been widely used in biology, agriculture, medicine and other fields.With the rise of SNP markers, their detection technologies have also developed rapidly. Currently, the primary SNP detection methods include gene chips (also known as DNA microarrays) [13], Cleaved Amplified Polymorphism Sequences (CAPS) [14], Single Strand Conformation Polymorphism (SSCP) [15], High-Resolution Melting (HRM) [16], Direct RNA Sequencing (DRS) [17], and the Penta-primer Amplification Refractory Mutation System (PARMS) [18]. The Penta-primer Amplification Refractory Mutation System (PARMS) is an SNP genotyping technology based on the four-primer amplification refractory mutation system [18]. The detection samples undergo a single polymerase chain reaction amplification and do not require electrophoresis detection [19]. A fluorescence scanner directly obtains the amplification data from the original plate.

This technology is similar to Competitive Allele Specific PCR (KASP), which detects different gene types by scanning two fluorescent signals at the end of a single sample using laser technology [20]. It offers advantages such as high throughput, high accuracy, good genetic stability, high flexibility, strong locus specificity, and low cost [21, 22].KASP genotyping technology is widely applied in crop breeding, enabling precise detection of specific genetic loci. This facilitates the improvement of agronomic traits, significantly accelerating the speed and efficiency of selection.

As far as we know, the literature on the development of mango SNP markers is still scarce. Although SNP markers, as the third-generation molecular markers, have been successfully applied to variety authenticity identification and hybrid progeny identification in a variety of crops, SSR, ISSR and AFLP are still the main molecular markers in mango research. There are problems such as low flux, high cost and unstable typing, especially in germplasm resource identification, genetic diversity analysis, fingerprint construction and purity identification of hybrid offspring. Through comprehensive comparative evaluation, PARMS technology shows superior applicability in the initial development stage of SNP markers compared with other methods. Therefore, this study was the first to use PARMS technology to establish a SNP marker system, systematically analyze the genetic diversity of mango germplasm resources and construct a fingerprint, marking the first systematic attempt in this research field.

The objectives of the present study were to: (1) identify uniformly distributed and highly polymorphic SNP loci in mangoes; (2) develop reliable KASP markers and screen highly polymorphic markers as core markers for constructing mango DNA fingerprinting profiles; (3) Utilize the aforementioned KASP markers to reveal genetic similarity among mango germplasm resources, construct DNA fingerprinting profiles, and provide a foundation for genetic research, variety identification, and purity assessment.

## Methods

### Plant material

The mango leaves were randomly collected from the ' Tropical Fruit Germplasm Resources Nursery of the Ministry of Agriculture ' (21° 10 ′ N, 110° 14 ′ E) of the South Subtropical Crops Research Institute of the Chinese Academy of Tropical Agricultural Sciences. The specific numbers of 145 collected materials are shown in Table 1, and they are grouped according to different uses. Among them, 6 materials numbered m7-m11 and m16 were used to screen primers for PARMS—SNP system. Three materials numbered m8, m9 and m16 were used for the construction and verification of the system. Forty-eight materials numbered m10-m57 were used for system verification. The 15 materials numbered m1-m15 were used for primer screening; all 145 (m2-m146) materials were used for accurate identification and analysis of germplasm resources.

**Table 1:** Varieties and serial numbers of mango tested

Code	Variety	Code	Variety	Code	Variety
m1	Kent	m27	Early Mango	m53	Baozaian
m2	Dongzhen Red Mango	m28	Hutou mang	m54	Thailand No.2
m3	Red Ivory	m29	Sri LanKa 811	m55	Pairi
m4	Ganhong	m30	200,007	m56	M. Sylvalica
m5	Golek	m31	Lilley	m57	Ai mang
m6	Renong No.1	m32	Tainong No.1	m58	Parrot mango
m7	Keitt	m33	Lüpimang	m59	Thailand 504
m8	Magovar	m34	Carabao	m60	200,001
m9	Zillate	m35	Lippens	m61	Haden
m10	Panxi hong mang	m36	Banganpalli	m62	Mallika
m11	Renong No.2	m37	Spooner	m63	200,002
m12	Big White Jade	m38	9401	m64	Hongmang No.11
m13	Guangxi 4	m39	Bambaroo	m65	Edward
m14	KRS	m40	1030	m66	Saigon
m15	R2E2	m41	Kensinting	m67	VanDyKe
m16	814	m42	Thailand shengshimang	m68	Glenn
m17	Guixiang mang	m43	Liuxiang mang	m69	Valencia Pride
m18	Ono	m44	Qinayia No. 1	m70	Guire No.82
m19	Pineapple Mango	m45	Qinayia No. 2	m71	Guifei1
m20	200,003	m46	Alphonso	m72	200,008
m21	200,004	m47	Putao mang	m73	Jian mang
m22	Machesu	m48	Xiangjao mang	m74	Fenshunwuhe mang
m23	Haibao Mang	m49	Thailand Carabao	m75	Xiaoqingpi
m24	Guire No.10	m50	Xiamao mang	m76	Cuba 1
m25	Thailand 506	m51	Thailand yeshengmang	m77	Cuba 2
m26	Chuanmang	m52	Guangxi 8	m78	Cuba 3
Code	Variety	Code	Variety	Code	Variety
m79	Cuba 4	m102	Guangxi 8	m125	115
m80	Okrong	m103	Huangxianya	m126	305
m81	Sijimang	m104	523	m127	AK
m82	Xiaojimang	m105	Fanghong	m128	301
m83	Feizhoudaxiangya	m106	Xingre 1	m129	412
m84	Gubasankeli	m107	Chowsa	m130	1002
m85	Siam Mango	m108	TA	m131	135–1
m86	Hongjinfeng	m109	524	m132	135–2
m87	Dayiwen	m110	Sannianmang	m133	521
m88	Yiwen	m111	Wugongci	m134	101
m89	Guifei 2	m112	Xingre 2	m135	703
m90	Yiwenqingpi	m113	Fu 1	m136	Dasannian
m91	Hongjinlong	m114	Fu 2	m137	1101
m92	Neelum	m115	Fu 3	m138	509
m93	Nang klangwan	m116	Red mango	m139	Duohua(Thailand Sijimang)
m94	Thai Red	m117	X	m140	HongJinHuang
m95	Small Philippine Mango	m118	Ba mang	m141	Hongwacheng
m96	Jingsuimang	m119	1601	m142	Ruby
m97	Zill	m120	Baiyumang	m143	Bombay Green
m98	Red Mango 8	m121	120	m144	Irwin
m99	Yuemang	m122	113	m145	JinHuang
m100	1506	m123	Huangyu	m146	Panyu No.4
m101	Naiyoumang	m124	1102		

This study used 318 F1 generation materials from the “Keitt” (KT) × “Renong No.1” (RN) cross and their parental lines as experimental materials. Among these, the 110 materials with Renong No.1” (RN) as the paternal line and “Keitt” (KT) as the maternal line were assigned to Group A; Group B consisted of 208 materials obtained with “Keitt” (KT) as the paternal line and “Renong No.1″ (RN) as the female parent.

### Primer design, synthesis and screening

Using the 33 mangos simplified genome sequencing reads obtained by the research group in the previous study [23] The reads were aligned to the NCBI mango reference genome GenBank GCA_016746415.1 [24] using the bwa software, and two methods were used to detect SNPs (Li et al., 2009). According to [25] standards, with slight modifications, perfect SNPs were screened using the following criteria: minimum allele frequency (MAF) > 0.4; mismatch rate < 0.25; heterozygosity rate < 0.5; no other mutations within 70 bp on either side of the SNP site. Primer design was performed using the online software SNP Primer (http://www.snpway.com). The 5' ends of primers X and Y were each added with adapters for fluorescent universal primer matching amplification.

Primer X was added with an adapter sequence matching FAM fluorescence: GAAGGTGACCAAGTTCATGCT. Primer Y had a primer sequence matching HEX fluorescence: GAAGGTCGGAGTCAACGGATT. The synthesized primers were synthesized by Sangon Biotech (Shanghai) Co., Ltd. Based on 33 SNP loci screened from simplified genome sequencing of mangoes, candidate SNP markers were synthesized using primers.

### Construction of fingerprint based on mango SNP core markers

Using the SNP markers developed in this study, core markers that meet the criteria were selected. Based on the core SNP markers, PARMS-SNP technology was used to genotype mango germplasm. Based on the gene types of each sample at the core SNP markers, a fingerprint database of mango germplasm resources based on core SNP markers was constructed.

### Hybrid authenticity identification of hybrid offspring

Using 318 F1-generation populations and parental lines of the “Kate” × “Re Nong 1” cross as materials. Based on the SNP markers developed in this study, the authenticity of the offspring of mango hybrid combinations was verified. First, the parents of the hybrid progeny were identified among the 145 mango materials used in the core marker screening. Next, the parents were screened for homozygous loci that differed in mango SNP markers, and these markers were used to identify the 318 hybrid progeny.

### Data statistics and analysis

Based on the fasta format file of the mango reference genome on NCBI and the chromosome positions and physical positions of the perfect SNPs sites screened in advance, the Tbtools software was used to extract sequences and draw a distribution map of SNP sites on the chromosome.

Using the Poppr and Ade4 packages in R, the data obtained from the ABI QuantStudio6 QS6 was organized and then subjected to principal component analysis and plotted. Using the Structure 2.3.4 software, 145 mango samples were divided into population groups, and the optimal cluster grouping. The number of genetic populations was set to K = 2–7, the Burn-in period was set to 100,000, the MCMC iteration count was set to 100,000, and each K value was calculated 10 times. The obtained Results folder containing the results compressed and uploaded to the Structure Harvester (https://taylor0.biology.ucla.edu/structureHarvester/) online website. The optimal K value was determined based on the LnP(D) value and the rate of change ΔK (by plotting a line graph of K and ΔK, the K value at which ΔK is maximum is the most appropriate K value).

## Results

### Screening and validation of SNP loci

**Fig. 1:**
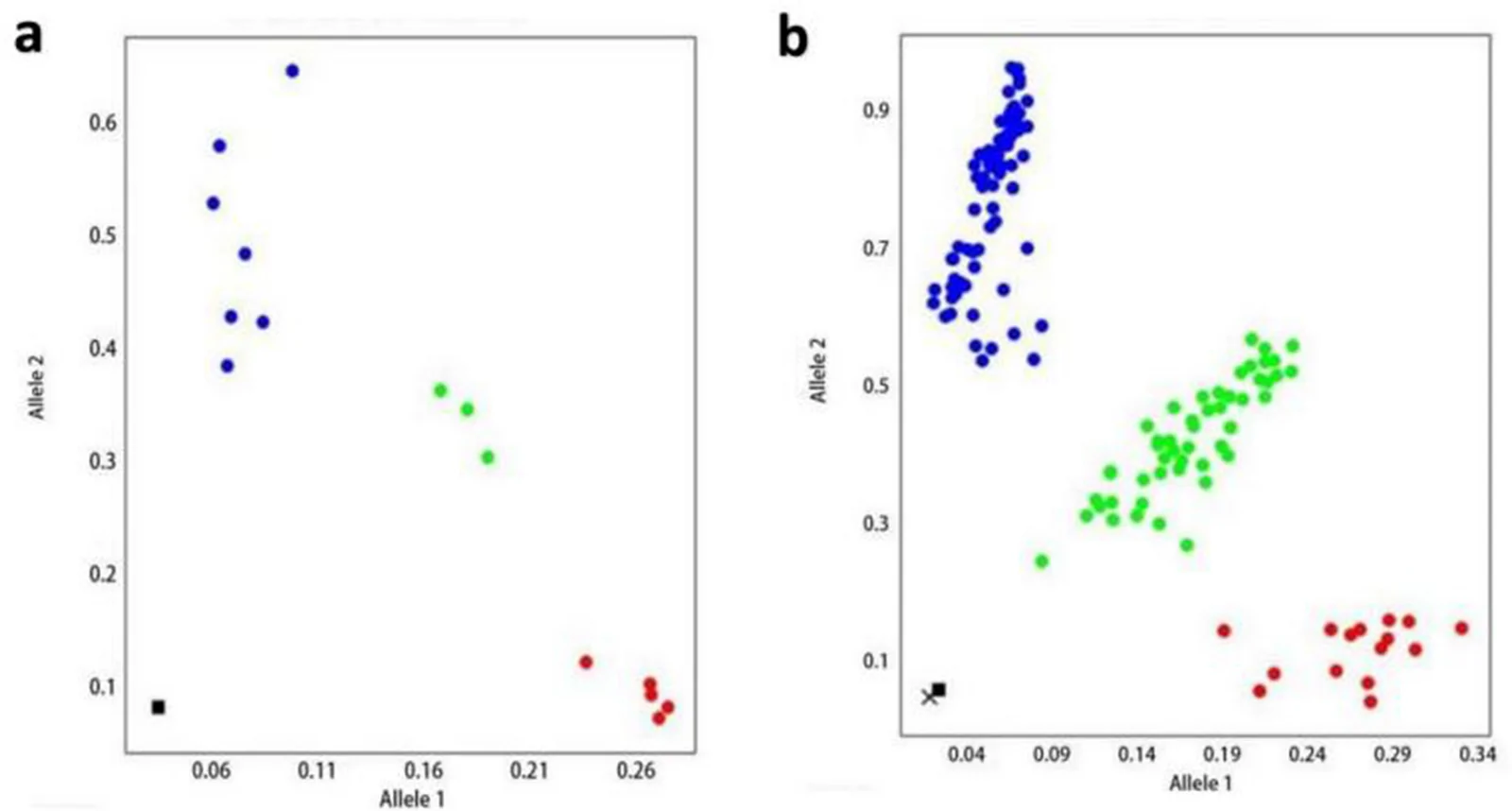
Schematic diagram of SNP-based genotyping of mango materials: **a** Schematic diagram of preliminary genotyping of 15 mango samples using SNP9; **b** Schematic diagram of genotyping of 145 mango samples using SNP120

This study is based on the simplified genome sequencing results of 33 mango varieties from the research team. After filtering with the GATK software, SNP-InDel information was obtained, resulting in a total of 438,322 SNP sites. According to the screening criteria, 272 high-quality SNP sites were obtained. Primers were designed for these 272 SNP sites, with a success rate of 93.4%. Among them, primers failed to be designed for 18 sites, while primers were successfully designed for the remaining 254 sites, which were identified as candidate markers. Finally, based on the SNP genotyping detection results, primers with good amplification efficiency and clear genotyping were selected. The genotyping results are shown in Fig. 1.

**Fig. 2:**
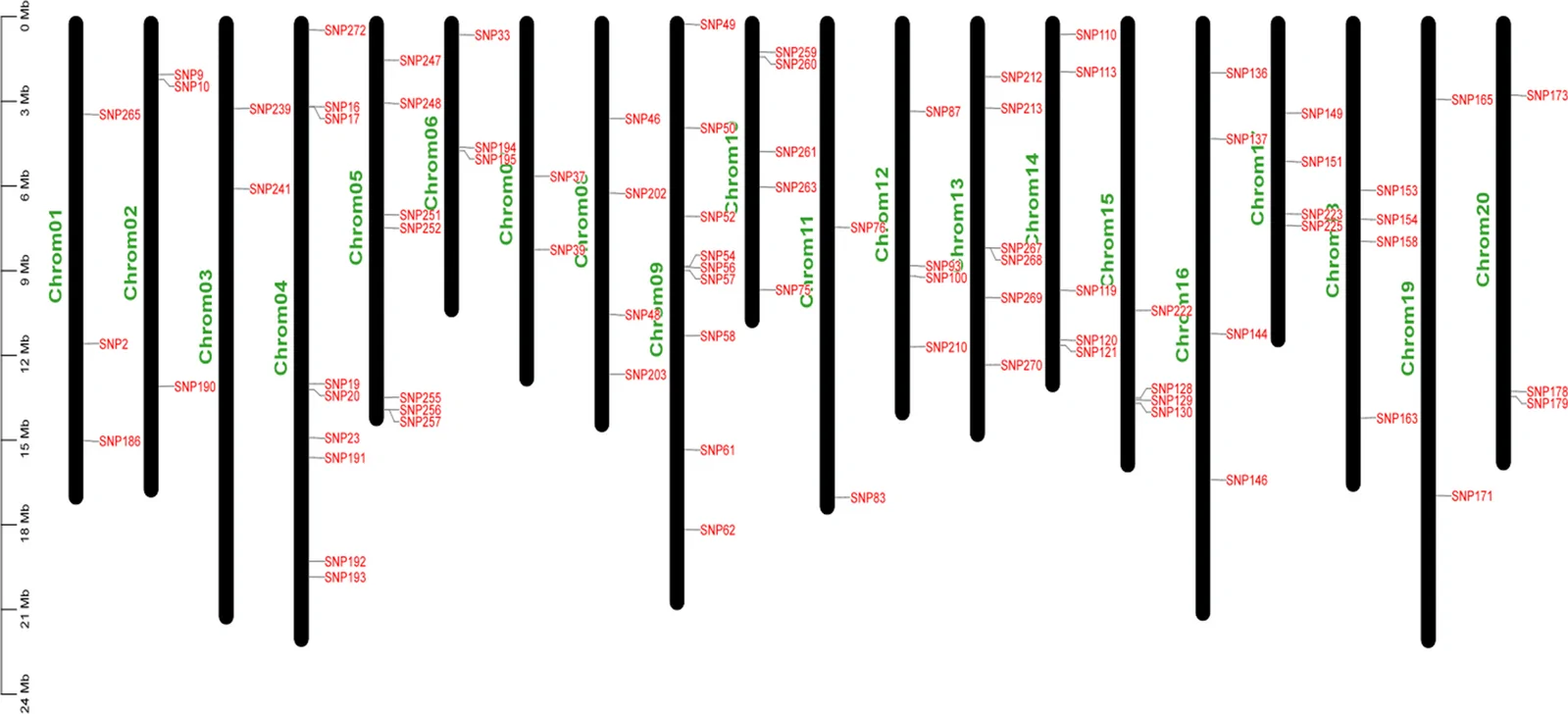
85 location of SNP markers on 20 chromosomes

Based on the results, SNP markers with better genotyping quality and a sample loss rate < 10% were selected. Ultimately, 85 novel SNP markers were identified from the 254 candidate SNP markers (Appendix A). As shown in Fig. 2, these 85 pairs of markers are distributed across each chromosome, with an average of 4 loci per chromosome. Chromosome 9 has the highest number of loci, with 9 SNP loci.

### SNP locus polymorphism analysis

**Fig. 3:**
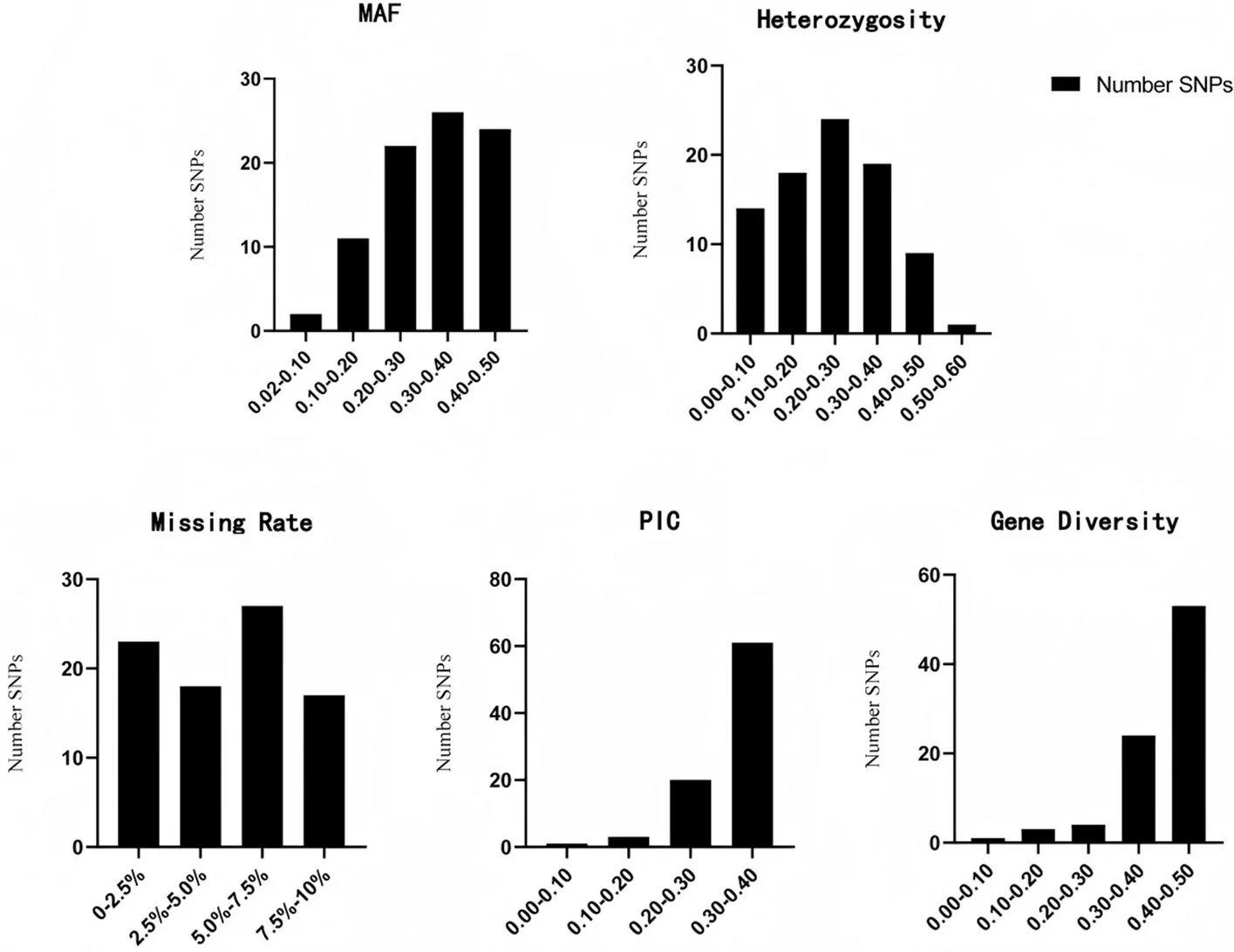
Genetic information obtained from 85 SNP markers based on 145 mango varieties

The genotype data of 85 markers were analyzed by PowerMarker 3.25 software (Appendix B). As shown in Fig. 3, the minor allele frequency (MAF) of most markers ranges between 0.2 and 0.5, with polymorphism information content (PIC) and gene diversity (Gene Diversity) between 0.3 and 0.5, indicating high genetic diversity. The heterozygosity of these markers generally follows a normal distribution, and the variation in the missing rate is relatively small, with most values below 7.5%.

### Structure and genetic diversity analysis of 145 mango based on SNP markers

**Fig. 4:**
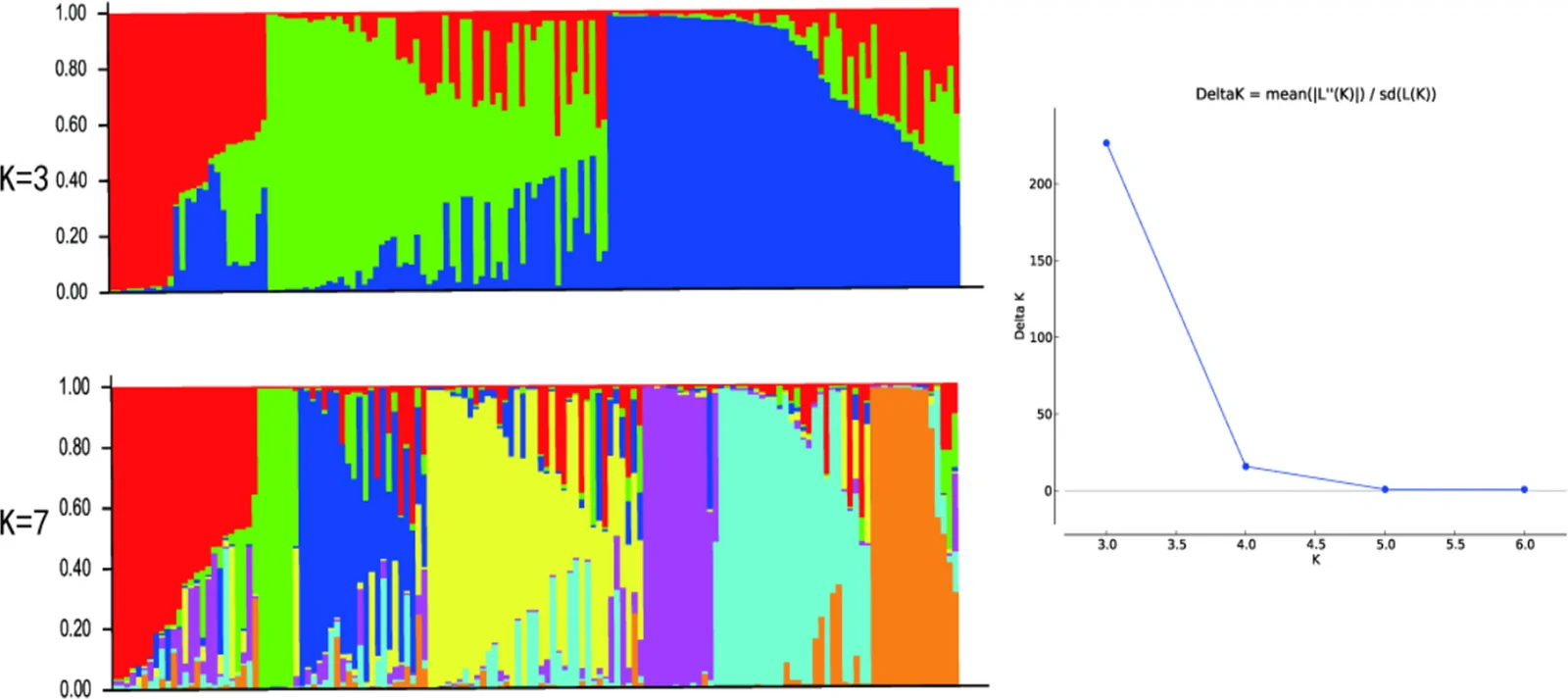
Group structure diagram and K-value and ∆K trend change diagram

Using Structure software to analyze the population genetic structure of 145 mango germplasm resources, the results (Fig. 4) indicate that when K = 3, the ∆K value is maximal, making it most reasonable to divide these 145 mango materials into three major clusters. Cluster A (red) contains 26 materials, Cluster B (green) contains 57 materials, and Cluster C (blue) contains 62 materials. The mixed colors within each cluster indicate the presence of genetic linkage and admixture between them.

**Fig. 5:**
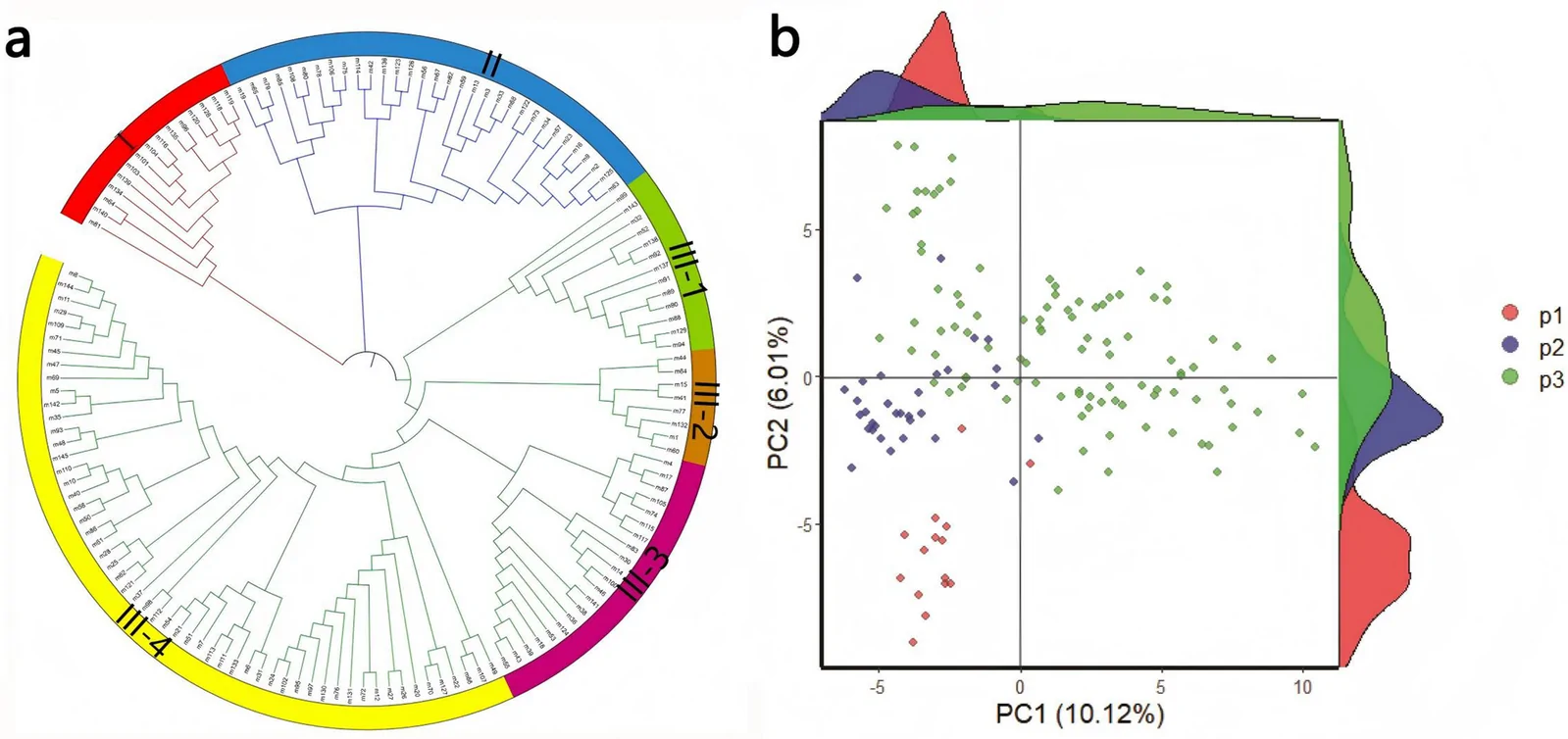
Cluster analysis of 145 mango varieties and Two-dimensional diagram of principal component analysis

Based on the cluster analysis results of 85 SNP markers (Fig. 5a), 145 mango germplasms could be divided into three major groups. Group I included 15 cultivars, such as 'Hongmang No.11 ' (m64), ' Sijimang ' (m81), ' Jingsuimang' (m96) and so on. Group II was composed of 32 varieties, mainly including ' Dongzhen Red Mango ' (m2), ' Red Ivory ' (m3), ' Zillate ' (m9) and so on. The varieties of this group were mostly from Thailand, the Philippines and China 's Yunnan and Guangxi regions, and most of them were polyembryonic. Group III contained the remaining 98 mango varieties, covering a variety of mainstream mango varieties, and could be further divided into 4 subgroups: In the III-1 subgroup, there were 13 germplasms including ' Tainong No.1 ' (m32), ' Guangxi 8 ' (m52) and so on, which were mainly derived from Taiwan and Sanya of China, and the fruits were mostly oval. There were only 8 germplasms in the III-2 subgroup, such as ' Kent ' (m1) and ' R2E2 ' (m15), mainly from Indonesia and Vietnam, and the fruit shape was mainly oval. Subgroup III-3 included 21 varieties, such as ' Ganhong ' (m4), ' KRS ' (m14), etc. Most of them were from Australia, and the embryo was mainly polyembryonic. Subgroup III-4 was composed of the remaining 56 germplasms from a wide range of sources, mainly including India in Southeast Asia, Florida and Cuba in North America, and some from Guinea and China in Africa. Most varieties in this subgroup are also polyembryonic types. In this subgroup, the germplasm with close genetic relationship showed obvious aggregation in clustering. For example, one of the parents of ' Mallika' (m62) was Neelun, while ' Guire No.82 ' (m70) was the seedling offspring of Neelun, and the clustering position of the two was close. Similarly, ' Renong No.2 ' (m11), ' Sri LanKa 811 ' (m29), ' Lippens ' (m35), ' Putao mang ' (m47) and ' Ruby ' (m142) were all ' Golek' (m5) or their offspring, which were closely related and clustered in the cluster analysis.

To validate the clustering analysis results, principal component analysis (PCA) was further performed on the genotyping data, and the results were consistent with the phylogenetic tree (Fig. 5 b). The first three principal components explained 10.12% (PC1) and 6.01% (PC2) of the total genetic variance, respectively. Compared with P1 and P2, P3 contained more clusters, reflecting the differing degrees of genetic diversity among subpopulations. Additionally, the overlapping points in the central regions of the three populations indicated the possible existence of gene exchange.

### Construction of fingerprint based on mango SNP core markers

**Fig. 6:**
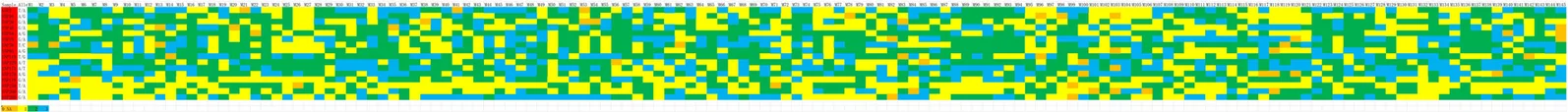
Fingerprint of 145 mango germplasm based on 16 pairs of core markers

According to the core primer screening criteria for the construction of KASP-SNP fingerprint of flowering Chinese cabbage [26], based on the four parameters of minor allele frequency (MAF) greater than 0.25, polymorphism information content (PIC) greater than 0.35, deletion rate less than 0.03, and heterozygosity rate less than 0.60, the core SNP markers were screened out, and the SNP fingerprint of 145 mango varieties was constructed. Among the 85 SNP markers, there were 85 markers with a heterozygosity rate less than 0.60, 23 markers with a deletion rate less than 0.03, and 58 markers with an MAF value greater than 0.25. There were 39 markers with PIC value greater than 0.35, and 16 markers met the above four conditions at the same time. These 16 markers(Appendix C) were used as core SNP markers. According to the core SNP markers and 145 mango varieties, a fingerprint database was constructed (Fig. 6). The results showed that the heterozygous genotype and the two homozygous genotypes were evenly distributed on the core SNP markers, indicating good genetic diversity. The fingerprint constructed by using the core SNP marker can effectively distinguish most mango varieties. However, the DNA fingerprint profiles of the three germplasm accessions, M95, M24, and M102, exhibit certain similarities, while the remaining 142 mango varieties possess unique and identifiable profiles.

The horizontal axis represents 145 germplasm accessions, while the vertical axis denotes 16 core primer pairs. Orange indicates absence, yellow represents gene 1, green denotes gene 2, and blue signifies heterozygous gene 3.

### Authenticity verification of mango hybrid offspring

DNA was extracted from the leaves of 318 F1 populations of mango “Keitt”(KT) × “Renong No.1”(RN) by CTAB method, and 85 SNP markers were screened. According to the polymorphism index, the minimum allele frequency, the quality of genotyping, the repeatability of the results and other indicators, three pairs of specific SNP primers for “Keitt”(KT) × “Renong No.1”(RN) were excavated. A total of 9 primer sequences were obtained using these three pairs of primers. Based on the SNP high-throughput technology platform, two groups of reciprocal cross materials were identified: group A included 110 F1 progeny samples of ' Renong No.1 ♂ × Keitt♀ ' and their parents and negative controls; group B included 208 F1 progeny samples of ' Keitt ♂ × Renong No.1 ♀ ' and their parents and negative controls. The results showed that these primers could effectively distinguish the parents and the control materials (Table 2).

**Table 2:** Identification of true and false hybrids of mango F_1_ generation (Group A)

编号	SNP49	SNP113	SNP272
KT(♀)	A:A	C:C	C:C
RN1(♂)	G:G	A:A	G:G
**F**_**1**_**-A-8**	**A:A**	**C:C**	**C:C**
**F**_**1**_**-A-37**	**A:A**	**C:C**	**C:C**
**F**_**1**_**-A-90**	**A:A**	**C:C**	**C:C**
F_1_-A-1	A:G	A:C	C:G
F_1_-A-2	A:G	A:C	C:G
F_1_-A-3	A:G	A:C	C:G
F_1_-A-4	A:G	A:C	C:G
F_1_-A-5	A:G	A:C	C:G
F_1_-A-6	A:G	A:C	C:G
F_1_-A-7	A:G	A:C	C:G
…	…	…	…
F_1_-A-109	A:G	A:C	C:G
F_1_-A-110	A:G	A:C	C:G

The bold entries indicate the F1 individuals identified as false hybrids by the SNP markers

**Fig. 7:**
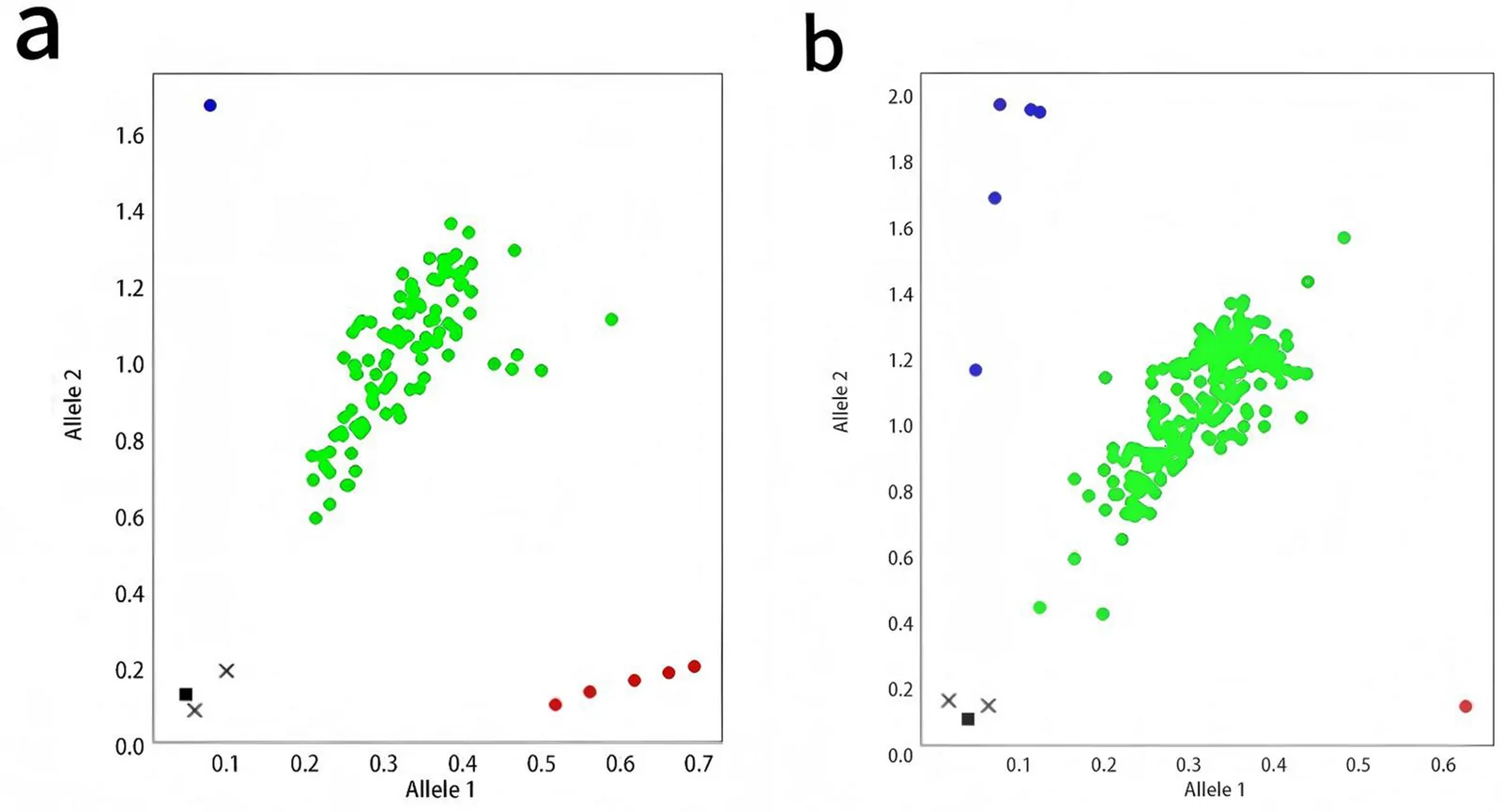
A schematic diagram of SNP marker for identification of true and false hybrids**. a** Typing map of “Keitt” (KT) ♀ × “Renong No.1” (RN) ♂ by primer SNP49; **b **Typing map of “Keitt” (KT) ♂ × “Renong No.1” (RN) ♀by primer SNP49

The results showed that the SNP49 marker could be clearly typed in the two populations, and the signal was stable and the discrimination was good. Figure 7a shows the identification results of group A: the negative control (ultrapure water) results were normal, two offspring samples were not detected, four offspring samples were the same as the female parent as red, one sample was the male parent genotype as blue, and the remaining 104 samples The genotype of the sample is concentrated in the middle of the parents. The position is green, which is identified as a true hybrid F1 generation.Fig. 7b shows the identification results of group B: the negative control (ultrapure water) was normal, two offspring samples were not detected, the genotypes of four offspring samples were the same as the female parent as blue, one sample was the male parent genotype as red, and the genotypes of the remaining 202 samples were concentrated in the middle of the parents. The position is green, which is identified as a true hybrid F1 generation.

Similarly, the authenticity of the hybrid seedlings detected by SNP 113 and SNP 272 was 93.4% and 91.2%, respectively. The details are shown in Table 3 (the thickened is the F1 generation material identified as false in the marker).

**Table 3:** Identification of true and false hybrids of mango F1 generation (Group B)

编号	SNP49	SNP113	SNP272
RN1(♀)	G:G	A:A	G:G
KT(♂)	A:A	C:C	C:C
**F**_**1**_**-B-1**	**G:G**	**A:A**	**G:G**
**F**_**1**_**-B-23**	**G:G**	**A:A**	**G:G**
**F**_**1**_**-B-28**	**G:G**	**A:A**	**G:G**
F_1_-B-2	A:G	A:C	C:G
F_1_-B-3	A:G	A:C	C:G
F_1_-B-4	A:G	A:C	C:G
F_1_-B-5	A:G	A:C	C:G
F_1_-B-6	A:G	A:C	C:G
F_1_-B-7	A:G	A:C	C:G
F_1_-B-8	A:G	A:C	C:G
…	…	…	…
F_1_-B-207	A:G	A:C	C:G
F_1_-B-208	A:G	A:C	C:G

The bold entries indicate the F1 individuals identified as false hybrids by the SNP markers

## Discussion

This study presents a novel high-throughput SNP genotyping approach utilizing genome-wide SNP markers to analyze genetic diversity, construct fingerprint profiles, and authenticate hybrid progeny within mango germplasm resources. Notably, for the first time, this research systematically developed genome-wide SNP markers for mango by innovatively screening high-quality SNP sites using PARMS technology. This development provides new tools for mango molecular breeding, variety identification, and assessment of genetic diversity. In comparison to conventional DNA molecular marker methods, the SNP markers introduced in this study exhibit higher throughput, reduced processing times, and lower costs, highlighting both technological innovation and operational efficiency.

In mango genetic diversity studies, traditional molecular markers, including SSR, SRAP, CDDP, and CEAP [6, 27–29], have been widely used. However, these approaches are often hindered by limitations such as a restricted number of markers, prolonged analysis times, and fewer detectable loci. In contrast, the present study employed SNP marker technology, combined with PARMS and high-throughput genotyping, to identify 272 high-quality SNP loci. Analysis of the resulting genotype data revealed the genetic background and phylogenetic relationships of 145 mango accessions, uncovering significant genetic diversity among populations. This approach represents a substantial advancement in mango genetic diversity research, offering robust data to support subsequent efforts in variety identification, breeding, and marker-assisted selection.

Furthermore, this study constructed a DNA fingerprinting profile for mango using SNP markers. Using a set of 16 highly polymorphic and informative core SNP markers, the profile enabled the discrimination of 142 out of 145 mango germplasm accessions, demonstrating the feasibility of this technique for cultivar identification. The SNP-based fingerprinting approach offers advantages such as simplified procedures, lower cost, and stable, accurate results. Therefore, it represents a reliable technique for cultivar identification and authenticity verification. Compared to traditional markers like SSRs [30], SNP markers provide higher resolution for distinguishing closely related cultivars, along with advantages in throughput, reproducibility, and automation, thereby significantly improving the precision and efficiency of identification. Consequently, SNP fingerprinting has considerable application potential in molecular identification, cultivar rights protection, and germplasm management of mango. Notably, in this study, mango cultivars M95(Small Philippine Mango), M24(Guire No.10), and M102(Guangxi 8) exhibited identical genotypes at the core SNP marker sites. However, combined with supplementary material 1, morphological analysis showed that there were significant differences in multiple traits of leaves and stems/branches. Regarding leaf traits, M95 exhibited the most prominent midrib, wavy leaf curl, and shorter petioles; M24 showed moderate midrib prominence and leaf curl with shorter petioles; M102 displayed flat leaves with smooth midribs and longer petioles. For stem and branch traits, M95 branches are slender with numerous apical branches; M24 branches are the shortest and most robust, exhibiting compact branching; M102 branches are of moderate length, displaying diverse apical branching patterns. These results indicate that, despite high molecular marker similarity among M95, M24, and M102, morphological differences enable the effective differentiation of these three varieties through their distinct morphological characteristics. Therefore, while existing core SNP markers effectively distinguish most mango cultivars, their discriminatory power is limited for certain varieties with similar genetic backgrounds that are less common in production. Future research should focus on developing more specific molecular markers to enhance the accuracy and reliability of cultivar identification. In hybrid progeny authenticity verification, traditional methods typically rely on phenotypic observation and SSR molecular markers, which often require considerable time to confirm hybrid purity and authenticity. In contrast, the SNP-based hybrid progeny authenticity verification method established in this study enables rapid genotyping analysis during the seedling stage, significantly enhancing verification accuracy and efficiency. By screening SNP loci with genetic variation and integrating genotyping results, it accurately distinguishes hybrid parents from progeny at an early stage, avoiding the time delays and operational complexities inherent in traditional methods. Currently, molecular studies on the authenticity verification of mango hybrid progeny remain scarce. Only Yao et al. [31] utilized SSR molecular markers to authenticate the hybridity of 184 seedlings derived from the cross between the mango cultivar Jin Huang as the maternal parent and the cultivar Aiven as the paternal parent. This study marks the first application of the SNP molecular marker system for verifying the purity of mango hybrids. It not only addresses gaps in molecular authentication research for mango hybrid progeny but also expands the practical application of SNP markers in mango breeding practices.

In summary, this study provides a precise and efficient molecular tool for genetic improvement, variety identification and molecular breeding of mango. Through the development and application of genome-wide SNP markers, it not only improves the accuracy of mango genetic analysis, but also significantly accelerates the process of variety identification and breeding. Combined with SNP markers and phenotypic data, it is expected to further promote the development of mango molecular breeding and improve the efficiency of variety improvement. This study not only provides an important theoretical support for the genome research and breeding practice of mango, but also provides a useful reference for the genetic research and breeding of other fruit crops.

## Conclusions

This study developed 85 novel SNP markers based on whole-genome data, detecting 251 alleles across 145 accessions with an average minor allele frequency of 0.32, heterozygosity of 0.24, and PIC value of 0.32. Utilising these markers, genetic diversity, population structure, principal component analysis, and cluster analysis were conducted on 145 mango germplasm accessions, resulting in their classification into three major groups. Further screening identified 16 stable, highly informative, and polymorphic core SNP molecular markers for mangoes, which were used to construct fingerprint profiles for all 145 germplasm accessions. This represents the first development and application of PARMS technology for SNP marker establishment in mangoes, specifically for genetic diversity analysis and fingerprinting of mango germplasm resources. This achievement will provide significant support for mango breeding programmes. Additionally, three pairs of specific primers suitable for authenticating hybrids between “Kate” and “Rennong No. 1” were identified from the 85 SNP markers. These provide reliable molecular tools and theoretical foundations for genetic improvement, variety authentication, and genetic diversity research in mango germplasm resources.

## Supplementary Information

Supplementary Material 1

## Data Availability

The simplified genome sequencing data for 33 mango germplasm resources generated in this study have been submitted to the Genome Sequence Archive (GSA), specifically to the Genome Sequence Archive (GSA) under the National Genome Data Center (NGDC) at the China National Center for Biological Information (CNCB), Chinese Academy of Sciences, with the accession number CRA036787 (https://ngdc.cncb.ac.cn/gsa/browse/CRA036787).

## Appendix

**Table 4:** 85 SNP marker primer sequences

Code	Names	Allele	Primer-X		Primer-Y	Primer Common
1	SNP2	T/A	GAAGGTGACCAAGTTCATGCTGAATAGAATTCCTGATCGTCTTTTGACA		GAAGGTCGGAGTCAACGGATTGAATAGAATTCCTGATCGTCTTTTGACT	TGCACTGAAGATCCATCTCTTGAT
2	SNP9	T/C	GAAGGTGACCAAGTTCATGCTCCGCTTAAGACAGATTAGGACTCC		GAAGGTCGGAGTCAACGGATTCCGCTTAAGACAGATTAGGACTCT	TGGATATCATTTAAACTAAAGAATATTGAACC
3	SNP10	C/T	GAAGGTGACCAAGTTCATGCTTCTGGAAAATAATTGAAGCAGACATT		GAAGGTCGGAGTCAACGGATTTCTGGAAAATAATTGAAGCAGACATC	GCGATTTCTTTCTTTGAATTTCTCTCA
4	SNP16	A/G	GAAGGTGACCAAGTTCATGCTGCCAAAACAGCTTCATGAAAACTTTAG		GAAGGTCGGAGTCAACGGATTGCCAAAACAGCTTCATGAAAACTTTAA	TAGATCCGAGAGTCCCGTATGTAA
5	SNP17	C/T	GAAGGTGACCAAGTTCATGCTAAGTGAATGATTTGAACCAAGGAT		GAAGGTCGGAGTCAACGGATTAAGTGAATGATTTGAACCAAGGAC	CAAAGTACTTTGCTAAATGCGCAT
6	SNP19	T/G	GAAGGTGACCAAGTTCATGCTTCCCATTTTAAAACATATTATTTCTGCG		GAAGGTCGGAGTCAACGGATTTCCCATTTTAAAACATATTATTTCTGCT	TGGGGAAGGACACAACTAATTCAT
7	SNP20	G/A	GAAGGTGACCAAGTTCATGCTATGATAACTGAAGATAAGAATTCATTTTTACTA		GAAGGTCGGAGTCAACGGATTATGATAACTGAAGATAAGAATTCATTTTTACTG	AAATAATGGCGACCTTGATCATCC
8	SNP23	A/G	GAAGGTGACCAAGTTCATGCTACAAAATTTTGAGAAGGGTGGAAAATTAG		GAAGGTCGGAGTCAACGGATTACAAAATTTTGAGAAGGGTGGAAAATTAA	CCTACCAAAGGTTTTGTGAAGCAA
9	SNP33	C/T	GAAGGTGACCAAGTTCATGCTCAAGAACCTTGACTCACATTTGACT		GAAGGTCGGAGTCAACGGATTCAAGAACCTTGACTCACATTTGACC	ACTCTAATTAGGACTGGACCCGAT
10	SNP37	G/A	GAAGGTGACCAAGTTCATGCTCCCTTTTGCCTAAAACAATGCCTA	GAAGGTCGGAGTCAACGGATTCCCTTTTGCCTAAAACAATGCCTG		ACCACATAGCATTGATGATACTCA
11	SNP39	A/T	GAAGGTGACCAAGTTCATGCTGGACACAAGACTTATGAGTTATTCCATT	GAAGGTCGGAGTCAACGGATTGGACACAAGACTTATGAGTTATTCCATA		TTTGGCAGATGAATTCATGACAGT
12	SNP46	T/C	GAAGGTGACCAAGTTCATGCTAGAAAACGCTCATAAGACAATCTC	GAAGGTCGGAGTCAACGGATTAGAAAACGCTCATAAGACAATCTT		ACTTTGACATGTCGCTACCATTTC
13	SNP48	G/A	GAAGGTGACCAAGTTCATGCTACAACTATGATAATCTCATCACTATTACTTA	GAAGGTCGGAGTCAACGGATTACAACTATGATAATCTCATCACTATTACTTG		TCTCGATATTACTTCAACCAAGAACA
14	SNP49	A/G	GAAGGTGACCAAGTTCATGCTTGTTGGTAGTCTCAAATCACATATG	GAAGGTCGGAGTCAACGGATTTGTTGGTAGTCTCAAATCACATATA		CAAACCCATCCTCAATTGTTGGAA
15	SNP50	C/T	GAAGGTGACCAAGTTCATGCTAGTTTGTTGCTTGGATCAAAAAGT	GAAGGTCGGAGTCAACGGATTAGTTTGTTGCTTGGATCAAAAAGC		CCATTATTGTCAACACCTCAACCC
16	SNP52	T/C	GAAGGTGACCAAGTTCATGCTGCTTTTGTATTGGGTGGAGAATTCC	GAAGGTCGGAGTCAACGGATTGCTTTTGTATTGGGTGGAGAATTCT		TATAAAGTCCTAACTGCCTCGCAA
17	SNP54	A/G	GAAGGTGACCAAGTTCATGCTGATGAGGACTGAGTCACTAAGTGG	GAAGGTCGGAGTCAACGGATTGATGAGGACTGAGTCACTAAGTGA		TGAGGAAATACACCCAACTCATTCT
18	SNP56	A/G	GAAGGTGACCAAGTTCATGCTCTTGGGCCCTAGCTTGTCG	GAAGGTCGGAGTCAACGGATTCTTGGGCCCTAGCTTGTCA		ATATTCAGTTGATCAAATAATGTCACGT
19	SNP57	A/G	GAAGGTGACCAAGTTCATGCTTGAGAAATATACTGTAGATGGACCG	GAAGGTCGGAGTCAACGGATTTGAGAAATATACTGTAGATGGACCA		GGTCGATGGAAAATTGTGCCATAT
20	SNP58	C/G	GAAGGTGACCAAGTTCATGCTGAAAGAGCTAGCTGCCCAAATCG	GAAGGTCGGAGTCAACGGATTGAAAGAGCTAGCTGCCCAAATCC		CGTTCACCTTTCCCCAATAAACTT
21	SNP61	C/T	GAAGGTGACCAAGTTCATGCTTGAACACCACCCTTATGCATATAAT	GAAGGTCGGAGTCAACGGATTTGAACACCACCCTTATGCATATAAC		TGCGTACATTTGTTTGTCTGAGAA
22	SNP62	T/A	GAAGGTGACCAAGTTCATGCTATGGATTTGCCTGCCCTCATTCA	GAAGGTCGGAGTCAACGGATTATGGATTTGCCTGCCCTCATTCT		TTTGCAAGCATCCTACCTTCCTAT
23	SNP75	G/A	GAAGGTGACCAAGTTCATGCTTGACTTCGGCCATGAAAATGAATA	GAAGGTCGGAGTCAACGGATTTGACTTCGGCCATGAAAATGAATG		TTACTAGCTATGACATCCCCACAC
24	SNP76	T/C	GAAGGTGACCAAGTTCATGCTACAACTAATAATTTGTGCAATTGTGATAC	GAAGGTCGGAGTCAACGGATTACAACTAATAATTTGTGCAATTGTGATAT		CAAGTAGGACATTCCCTTCATCCA
25	SNP83	T/C	GAAGGTGACCAAGTTCATGCTCTTGTGGAAGCTAAAGAGATATAAGC	GAAGGTCGGAGTCAACGGATTCTTGTGGAAGCTAAAGAGATATAAGT		AGAATAGTCACTTATGGTGCCTCG
26	SNP87	A/C	GAAGGTGACCAAGTTCATGCTGCTGAATATAACACAAGAAGTCATGC	GAAGGTCGGAGTCAACGGATTGCTGAATATAACACAAGAAGTCATGA		AGACTCAGGAACCCATTGAAATAGA
27	SNP93	A/G	GAAGGTGACCAAGTTCATGCTAAAGCCCCCATACTATTGTCATTG	GAAGGTCGGAGTCAACGGATTAAAGCCCCCATACTATTGTCATTA		AACTTAGAGACCCCAAGGAGAAAG
28	SNP100	G/A	GAAGGTGACCAAGTTCATGCTGCAAGTGATAAGACTGCATAATGCTA	GAAGGTCGGAGTCAACGGATTGCAAGTGATAAGACTGCATAATGCTG		TGGGCATAAAATAAACATAAAAGACTAAGT
29	SNP110	A/G	GAAGGTGACCAAGTTCATGCTAGACGACATATAAATGAGGTTGGG	GAAGGTCGGAGTCAACGGATTAGACGACATATAAATGAGGTTGGA		TCAGACAAATACTCAAGAGTTCAATCA
30	SNP113	A/C	GAAGGTGACCAAGTTCATGCTTGAACACCACCCTTATGCATATAAT	GAAGGTCGGAGTCAACGGATTTGAACACCACCCTTATGCATATAAC		TGCGTACATTTGTTTGTCTGAGAA
31	SNP119	T/G	GAAGGTGACCAAGTTCATGCTGAACCTCATGTTGGCGCCATG	GAAGGTCGGAGTCAACGGATTGAACCTCATGTTGGCGCCATT		CGTTTTAAATGTTCGCTTTGGCAG
32	SNP120	C/T	GAAGGTGACCAAGTTCATGCTTCTTGGTTTATCCATTTTAAGCCGAT	GAAGGTCGGAGTCAACGGATTTCTTGGTTTATCCATTTTAAGCCGAC		TGGTGCCTCCAATGTTACATAGAA
33	SNP121	T/C	GAAGGTGACCAAGTTCATGCTAAAAACAACTTGGTCAGAGCTATC	GAAGGTCGGAGTCAACGGATTAAAAACAACTTGGTCAGAGCTATT		TGTGGATTCTAATACCTGTTTCAACA
34	SNP128	A/G	GAAGGTGACCAAGTTCATGCTCTTTGGGCTACCTGTGGCTTAG	GAAGGTCGGAGTCAACGGATTCTTTGGGCTACCTGTGGCTTAA		TAGGCTTTACCAACTCATGCATGA
35	SNP129	A/T	GAAGGTGACCAAGTTCATGCTAAGGCGACCTCAATTAACTTGCTT	GAAGGTCGGAGTCAACGGATTAAGGCGACCTCAATTAACTTGCTA		GGTACTTAGCATACCTAGCTCATGT
36	SNP130	A/T	GAAGGTGACCAAGTTCATGCTCATACTTGGCTTGGTTGAGCAAAT	GAAGGTCGGAGTCAACGGATTCATACTTGGCTTGGTTGAGCAAAA		AGCTTAACTATTTCCTTGGACTACA
37	SNP136	T/A	GAAGGTGACCAAGTTCATGCTGGCAGTTATGAGAAGGAGTTGGA	GAAGGTCGGAGTCAACGGATTGGCAGTTATGAGAAGGAGTTGGT		TCAATGAGGAGGCAGTTTGGATAA
38	SNP137	T/C	GAAGGTGACCAAGTTCATGCTGCTCATCTTATAACTAAACCTTGGAATAAAC	GAAGGTCGGAGTCAACGGATTGCTCATCTTATAACTAAACCTTGGAATAAAT		TACGCTCAGAATGGGGAAAGAATT
39	SNP144	C/G	GAAGGTGACCAAGTTCATGCTCACCTGTTCAAACTCAAATTTCCTTG	GAAGGTCGGAGTCAACGGATTCACCTGTTCAAACTCAAATTTCCTTC		AGCTCGAAATTGAATTCCAAGACG
40	SNP146	G/T	GAAGGTGACCAAGTTCATGCTCCCACACTTCCTTGCTTGTGT	GAAGGTCGGAGTCAACGGATTCCCACACTTCCTTGCTTGTGG		CTTTATCAAGGATTGGACAATGACA
41	SNP149	C/T	GAAGGTGACCAAGTTCATGCTACATATGGTCATATAGGGTAGATGCAT	GAAGGTCGGAGTCAACGGATTACATATGGTCATATAGGGTAGATGCAC		ATGGATCTGAGTATAGGGGAGTGT
42	SNP151	C/T	GAAGGTGACCAAGTTCATGCTGGTTCAAAACCCAATGAATTTAAAAACT	GAAGGTCGGAGTCAACGGATTGGTTCAAAACCCAATGAATTTAAAAACC		CCCCATGGTTCTATGGCATCATAA
43	SNP153	G/A	GAAGGTGACCAAGTTCATGCTCGCCGGCCTGTAAGTACA	GAAGGTCGGAGTCAACGGATTCGCCGGCCTGTAAGTACG		GAACACTCCGAAGAAGTGGATAGG
44	SNP154	C/G	GAAGGTGACCAAGTTCATGCTTAATCAGGGGAGGTGGGGAG	GAAGGTCGGAGTCAACGGATTTAATCAGGGGAGGTGGGGAC		CGAACTAAACATCCCCATCCAAAC
45	SNP158	T/C	GAAGGTGACCAAGTTCATGCTGAATTCTTGATGTAATTTGTAGAGAATTTTC	GAAGGTCGGAGTCAACGGATTGAATTCTTGATGTAATTTGTAGAGAATTTTT		GATCCTTTCTCGTGAGTATTCGGA
46	SNP163	T/C	GAAGGTGACCAAGTTCATGCTACATACTTAAATCAATGTTTGTAGTTGATC	GAAGGTCGGAGTCAACGGATTACATACTTAAATCAATGTTTGTAGTTGATT		AGGAAATGTTGATTTTGTACTTGTCT
47	SNP165	A/T	GAAGGTGACCAAGTTCATGCTATTCAACAACATCTTGTGAAGCTT	GAAGGTCGGAGTCAACGGATTATTCAACAACATCTTGTGAAGCTA		TTTAACTAGAAAGGGTGGCAGTGG
48	SNP171	A/T	GAAGGTGACCAAGTTCATGCTATCCCGTATGTACACAGCTCTATT	GAAGGTCGGAGTCAACGGATTATCCCGTATGTACACAGCTCTATA		GCTTGACTCAGTTCAACTTCACTC
49	SNP173	A/G	GAAGGTGACCAAGTTCATGCTCCGTCTCCGATGGCATTAG	GAAGGTCGGAGTCAACGGATTCCGTCTCCGATGGCATTAA		ATAACCTACGTCAATGGTTGGGAA
50	SNP178	A/G	GAAGGTGACCAAGTTCATGCTCAGGGATTGTACCAGTATAACATTTTG	GAAGGTCGGAGTCAACGGATTCAGGGATTGTACCAGTATAACATTTTA		GAAGATCCCATCACCCAGTAGATC
51	SNP179	G/A	GAAGGTGACCAAGTTCATGCTGCTAAGGGACGACATCTCTAATGAAA	GAAGGTCGGAGTCAACGGATTGCTAAGGGACGACATCTCTAATGAAG		GGGCCTCTTTGGAGAAGATAGTTA
52	SNP186	A/C	GAAGGTGACCAAGTTCATGCTTGGTATTGATTCTACCAAACAAAATAAC	GAAGGTCGGAGTCAACGGATTTGGTATTGATTCTACCAAACAAAATAAA		CCTTACAATGGCTTGGAATGTGTT
53	SNP190	T/C	GAAGGTGACCAAGTTCATGCTAGGTTTAATTTCAATTATTCCAGAATTCAAC	GAAGGTCGGAGTCAACGGATTAGGTTTAATTTCAATTATTCCAGAATTCAAT		GGTGTTTGACAGTTCATGCAAGTA
54	SNP191	T/G	GAAGGTGACCAAGTTCATGCTTGACAATCAATTTCTTGGATTTTAGGTG	GAAGGTCGGAGTCAACGGATTTGACAATCAATTTCTTGGATTTTAGGTT		AGGAATTTTCTTGGGTTACAACACT
55	SNP192	C/T	GAAGGTGACCAAGTTCATGCTCAATCCTATCCAGTTTCCAAATCCT	GAAGGTCGGAGTCAACGGATTCAATCCTATCCAGTTTCCAAATCCC		TGCAGATCTTGTTTCAGGCTATTG
56	SNP193	G/A	GAAGGTGACCAAGTTCATGCTTGAAAAAGTCATACACAAGTGAACCTAA	GAAGGTCGGAGTCAACGGATTTGAAAAAGTCATACACAAGTGAACCTAG		TGAGTTTTCCAATGATCCGACAAC
57	SNP194	T/C	GAAGGTGACCAAGTTCATGCTGCTTCGGCTTTATTGTATCTCCAC	GAAGGTCGGAGTCAACGGATTGCTTCGGCTTTATTGTATCTCCAT		GATATCTCTGTGGACAACGCATTG
58	SNP195	G/A	GAAGGTGACCAAGTTCATGCTACCCTTATATGTCATCAACATTGTTCATA	GAAGGTCGGAGTCAACGGATTACCCTTATATGTCATCAACATTGTTCATG		ACGTTAATATGGGATAAGTGAGAGA
59	SNP202	T/C	GAAGGTGACCAAGTTCATGCTGCATTGAATGTCTTTTATATGAGTAAGCC	GAAGGTCGGAGTCAACGGATTGCATTGAATGTCTTTTATATGAGTAAGCT		AACAATTTTCACCAACTCCCCTTG
60	SNP203	T/A	GAAGGTGACCAAGTTCATGCTTCAACGAATTCATAGTTTGTCATCATATTTA	GAAGGTCGGAGTCAACGGATTTCAACGAATTCATAGTTTGTCATCATATTTT		CCCATGGTCAACAACATTGTGTAT
61	SNP210	C/T	GAAGGTGACCAAGTTCATGCTAGTTGTCTTTTCAGATTTTATGTATTTGGAT	GAAGGTCGGAGTCAACGGATTAGTTGTCTTTTCAGATTTTATGTATTTGGAC		TCAATTGTTCAAAGTGTGTTGCAA
62	SNP212	T/A	GAAGGTGACCAAGTTCATGCTTCTGAGCCTTAAATTCCCCTTTCATA	GAAGGTCGGAGTCAACGGATTTCTGAGCCTTAAATTCCCCTTTCATT		TCACACACAATAAATGACATCTATACA
63	SNP213	G/A	GAAGGTGACCAAGTTCATGCTTGGAATTCCTAAGAAATGAAATACCCA	GAAGGTCGGAGTCAACGGATTTGGAATTCCTAAGAAATGAAATACCCG		TGGGGATTTTGTGAAGAAATTTGTCT
64	SNP222	G/A	GAAGGTGACCAAGTTCATGCTTGAGAACTAGAATCACTATGTCCTCTA	GAAGGTCGGAGTCAACGGATTTGAGAACTAGAATCACTATGTCCTCTG		TGTAGTTCATCAAATCGTGCAAGG
65	SNP223	T/C	GAAGGTGACCAAGTTCATGCTTCTTTGTTAACCACATTTTTATCATAATGC	GAAGGTCGGAGTCAACGGATTTCTTTGTTAACCACATTTTTATCATAATGT		TATCTTTAGGGCTCAAAGGCTTGA
66	SNP225	C/T	GAAGGTGACCAAGTTCATGCTAAATAAACAATGTGTATATCCATAAACACT	GAAGGTCGGAGTCAACGGATTAAATAAACAATGTGTATATCCATAAACACC		ACACAACGTTAGAATATCAAAGAATTTG
67	SNP239	A/C	GAAGGTGACCAAGTTCATGCTAAATAGAAAGGTATTTATGTATGAAAGGATC	GAAGGTCGGAGTCAACGGATTAAATAGAAAGGTATTTATGTATGAAAGGATA		TGCCTTACTACGTCATTGGGTAAA
68	SNP241	G/A	GAAGGTGACCAAGTTCATGCTAGGCTTAGGGTTAAGGTCATATAGA	GAAGGTCGGAGTCAACGGATTAGGCTTAGGGTTAAGGTCATATAGG		GTGATGTTTTATCAAGTTGCACGC
69	SNP247	T/C	GAAGGTGACCAAGTTCATGCTTGGTGCCAAATATTGTGACTCAAC	GAAGGTCGGAGTCAACGGATTTGGTGCCAAATATTGTGACTCAAT		CGAATTCCAATTTCCCTTGAACCA
70	SNP248	G/A	GAAGGTGACCAAGTTCATGCTTGTTCGGACGTATAACTCTTGCTA	GAAGGTCGGAGTCAACGGATTTGTTCGGACGTATAACTCTTGCTG		TGCCCAACATTCTTCCCTATTGAT
71	SNP251	C/T	GAAGGTGACCAAGTTCATGCTTGCATAACTACAAGAACAACCATTT	GAAGGTCGGAGTCAACGGATTTGCATAACTACAAGAACAACCATTC		TGAGCTAAACAGCTTATGCAAACC
72	SNP252	C/T	GAAGGTGACCAAGTTCATGCTCCAGCTACCGCTATACCAACTTTT	GAAGGTCGGAGTCAACGGATTCCAGCTACCGCTATACCAACTTTC		GCAAGAGTAAGCCCAAATCTGTTT
73	SNP255	A/G	GAAGGTGACCAAGTTCATGCTCCCTCTGGTGTCCAAAATCCAG	GAAGGTCGGAGTCAACGGATTCCCTCTGGTGTCCAAAATCCAA		TGATGGACAATTGCAGTCTTTAGC
74	SNP256	C/G	GAAGGTGACCAAGTTCATGCTTCCCTTGAATATTCGAATTCAGGTTG	GAAGGTCGGAGTCAACGGATTTCCCTTGAATATTCGAATTCAGGTTC		CTGTTGGAGAATATGACCAAACCG
75	SNP257	T/C	GAAGGTGACCAAGTTCATGCTAGTGAATCTATTGTTTGCATATACAAAGC	GAAGGTCGGAGTCAACGGATTAGTGAATCTATTGTTTGCATATACAAAGT		TTTGCACACATGGGTTAGTTTACC
76	SNP259	T/A	GAAGGTGACCAAGTTCATGCTTCTGAATACATGGGATAAGTGGCA	GAAGGTCGGAGTCAACGGATTTCTGAATACATGGGATAAGTGGCT		TGTAGGGATGCAATGAAAAGAGGA
77	SNP260	G/A	GAAGGTGACCAAGTTCATGCTAAATAGGGGTGGATTCGGGTCAA	GAAGGTCGGAGTCAACGGATTAAATAGGGGTGGATTCGGGTCAG		CGAACTCAAGCTGAATTCAAACCT
78	SNP261	T/C	GAAGGTGACCAAGTTCATGCTCGAAATCAATTTATAGAGCTATTTGTATTGC	GAAGGTCGGAGTCAACGGATTCGAAATCAATTTATAGAGCTATTTGTATTGT		GATCAAGGTCTGTTTTCTCCGTTG
79	SNP263	G/A	GAAGGTGACCAAGTTCATGCTGCAACACAATTTTGTTATAACGATCCAA	GAAGGTCGGAGTCAACGGATTGCAACACAATTTTGTTATAACGATCCAG		TAAAGCTTTGAGGTCTGATCGAGG
80	SNP265	G/T	GAAGGTGACCAAGTTCATGCTAGTTGGTGAGGCAAGTATCTTTTT	GAAGGTCGGAGTCAACGGATTAGTTGGTGAGGCAAGTATCTTTTG		ACTACATGCATCAATACAGGACGA
81	SNP267	A/T	GAAGGTGACCAAGTTCATGCTGGAACCTCTCTTGTAAAATAATATGCTTTT	GAAGGTCGGAGTCAACGGATTGGAACCTCTCTTGTAAAATAATATGCTTTA		ACAGTTTCAGGAAATATGCCCTCT
82	SNP268	G/A	GAAGGTGACCAAGTTCATGCTAGGGCATATTTCCTGAAACTGTAAATA	GAAGGTCGGAGTCAACGGATTAGGGCATATTTCCTGAAACTGTAAATG		GCTTCTGCATTGCATTTTCAAGAA
83	SNP269	G/C	GAAGGTGACCAAGTTCATGCTTCTAGTTAGTGACGTAGGCTTGAC	GAAGGTCGGAGTCAACGGATTTCTAGTTAGTGACGTAGGCTTGAG		CTAAACCTATGAGGCCTACCAAGT
84	SNP270	C/T	GAAGGTGACCAAGTTCATGCTTTCAGATCAGTTTCTGTAATCCCT	GAAGGTCGGAGTCAACGGATTTTCAGATCAGTTTCTGTAATCCCC		AGGATTAACAAGATGGAGGGCAAT
85	SNP272	C/G	GAAGGTGACCAAGTTCATGCTGGTGCGCTCTTTCATTCTGTAG	GAAGGTCGGAGTCAACGGATTGGTGCGCTCTTTCATTCTGTAC		TCTTCTCAAATTTCTAGGGTTAGGGT

The name of the marker labeled red is the core marker selected in this study

**Table 5:** Genetic information of 145 mango varieties on 85 SNP markers

Cord	Maker	Allele X/Y	Minor.Allele.Frquency	Gene Diversity	Heterozygosity	PIC	Missing rate
1	SNP2	T/A	0.47	0.50	0.39	0.37	0.02
2	SNP9	T/C	0.40	0.48	0.20	0.36	0.09
3	SNP10	C/T	0.34	0.45	0.36	0.35	0.03
4	SNP16	A/G	0.43	0.49	0.13	0.37	0.03
5	SNP17	C/T	0.33	0.44	0.20	0.35	0.08
6	SNP19	T/G	0.24	0.37	0.38	0.30	0.01
7	SNP20	G/A	0.48	0.50	0.11	0.37	0.03
8	SNP23	A/G	0.30	0.42	0.38	0.33	0.07
9	SNP33	C/T	0.38	0.47	0.25	0.36	0.04
10	SNP37	G/A	0.50	0.50	0.22	0.37	0.07
11	SNP39	A/T	0.38	0.47	0.05	0.36	0.06
12	SNP46	T/C	0.50	0.50	0.33	0.38	0.05
13	SNP48	G/A	0.49	0.50	0.27	0.37	0.02
14	SNP49	A/G	0.26	0.39	0.32	0.31	0.01
15	SNP50	C/T	0.23	0.35	0.23	0.29	0.08
16	SNP52	T/C	0.32	0.44	0.27	0.34	0.08
17	SNP54	A/G	0.36	0.46	0.06	0.35	0.02
18	SNP56	A/G	0.25	0.37	0.10	0.30	0.06
19	SNP57	A/G	0.33	0.44	0.20	0.34	0.06
20	SNP58	C/G	0.23	0.36	0.23	0.29	0.09
21	SNP61	C/T	0.36	0.46	0.32	0.35	0.06
22	SNP62	T/A	0.21	0.34	0.19	0.28	0.08
23	SNP75	G/A	0.42	0.49	0.38	0.37	0.02
24	SNP76	T/C	0.40	0.48	0.24	0.36	0.03
25	SNP83	T/C	0.22	0.35	0.15	0.29	0.08
26	SNP87	A/C	0.46	0.50	0.00	0.37	0.07
27	SNP93	A/G	0.39	0.48	0.11	0.36	0.01
28	SNP100	G/A	0.22	0.34	0.43	0.28	0.06
29	SNP110	A/G	0.22	0.34	0.20	0.28	0.07
30	SNP113	A/C	0.36	0.46	0.48	0.35	0.06
31	SNP119	T/G	0.43	0.49	0.33	0.37	0.03
32	SNP120	C/T	0.27	0.40	0.34	0.32	0.01
33	SNP121	T/C	0.41	0.48	0.28	0.37	0.05
34	SNP128	A/G	0.11	0.19	0.02	0.18	0.02
35	SNP129	A/T	0.38	0.47	0.22	0.36	0.02
36	SNP130	A/T	0.30	0.42	0.13	0.33	0.08
37	SNP136	T/A	0.28	0.41	0.04	0.32	0.06
38	SNP137	T/C	0.31	0.43	0.33	0.34	0.07
39	SNP144	C/G	0.22	0.34	0.08	0.28	0.08
40	SNP146	G/T	0.15	0.26	0.22	0.23	0.08
41	SNP149	C/T	0.21	0.33	0.02	0.27	0.08
42	SNP151	C/T	0.40	0.48	0.23	0.36	0.06
43	SNP153	G/A	0.26	0.38	0.16	0.31	0.03
44	SNP154	C/G	0.39	0.47	0.46	0.36	0.08
45	SNP158	T/C	0.25	0.37	0.31	0.30	0.03
46	SNP163	T/C	0.19	0.31	0.04	0.26	0.06
47	SNP165	A/T	0.17	0.28	0.11	0.24	0.06
48	SNP171	A/T	0.45	0.50	0.49	0.37	0.01
49	SNP173	A/G	0.27	0.39	0.00	0.31	0.04
50	SNP178	A/G	0.39	0.48	0.35	0.36	0.03
51	SNP179	G/A	0.44	0.49	0.18	0.37	0.01
52	SNP186	A/C	0.31	0.43	0.19	0.34	0.06
53	SNP190	T/C	0.43	0.49	0.55	0.37	0.09
54	SNP191	T/G	0.19	0.31	0.25	0.26	0.06
55	SNP192	C/T	0.49	0.50	0.12	0.37	0.06
56	SNP193	G/A	0.19	0.31	0.32	0.26	0.02
57	SNP194	T/C	0.31	0.43	0.48	0.34	0.06
58	SNP195	G/A	0.47	0.50	0.23	0.37	0.06
59	SNP202	T/C	0.03	0.05	0.06	0.05	0.01
60	SNP203	T/A	0.23	0.35	0.25	0.29	0.07
61	SNP210	C/T	0.33	0.44	0.36	0.35	0.03
62	SNP212	T/A	0.10	0.18	0.20	0.17	0.05
63	SNP213	G/A	0.30	0.42	0.36	0.33	0.03
64	SNP222	G/A	0.20	0.32	0.10	0.27	0.07
65	SNP223	T/C	0.47	0.50	0.00	0.37	0.10
66	SNP225	C/T	0.15	0.26	0.27	0.22	0.09
67	SNP239	A/C	0.30	0.42	0.24	0.33	0.04
68	SNP241	G/A	0.34	0.45	0.13	0.35	0.05
69	SNP247	T/C	0.19	0.30	0.08	0.26	0.01
70	SNP248	G/A	0.37	0.46	0.17	0.36	0.04
71	SNP251	C/T	0.09	0.16	0.11	0.15	0.05
72	SNP252	C/T	0.41	0.48	0.45	0.37	0.04
73	SNP255	A/G	0.47	0.50	0.47	0.37	0.08
74	SNP256	C/G	0.38	0.47	0.09	0.36	0.06
75	SNP257	T/C	0.44	0.49	0.11	0.37	0.06
76	SNP259	T/A	0.42	0.49	0.49	0.37	0.02
77	SNP260	G/A	0.41	0.48	0.14	0.37	0.03
78	SNP261	T/C	0.42	0.49	0.17	0.37	0.06
79	SNP263	G/A	0.15	0.26	0.31	0.23	0.06
80	SNP265	G/T	0.20	0.32	0.23	0.27	0.06
81	SNP267	A/T	0.41	0.48	0.27	0.37	0.10
82	SNP268	G/A	0.49	0.50	0.37	0.37	0.01
83	SNP269	G/C	0.39	0.48	0.41	0.36	0.05
84	SNP270	C/T	0.19	0.31	0.34	0.26	0.05
85	SNP272	C/G	0.26	0.39	0.24	0.31	0.05

**Table 6:** Fingerprints of 145 mango varieties based on 16 SNP markers

Sample	S-2	S-16	S-20	S-48	S-54	S-75	S-76	S-93	S-119	S-129	S-171	S-178	S-179	S-259	S-260	S-268
Allele 1/2	T/A	A/G	G/A	G/A	A/G	G/A	T/C	A/G	T/G	A/T	A/T	A/G	G/A	T/A	G/A	G/A
m1	AA	AG	AA	GG	GG	GA	NA	GG	TG	AA	AA	AA	GG	TT	GG	GG
m2	TA	GG	AA	AA	GG	AA	CC	GG	TG	AA	AA	AG	GG	TT	GG	AA
m3	TT	AA	GA	AA	GG	AA	NA	GG	GG	AA	AT	AG	GG	TT	GG	GA
m4	TT	AA	AA	GG	AG	GG	TC	GG	TT	TT	AT	AA	GA	TT	AA	GG
m5	AA	GG	GG	GA	AA	AA	TC	GG	TT	TT	AT	AG	NA	TT	GA	GA
m6	AA	GG	GA	GA	AA	AA	CC	GG	TT	AT	TT	GG	GG	TT	GG	GG
m7	AA	GG	AA	AA	AA	AA	TC	AG	TT	TT	AT	GG	GG	TT	GG	GG
m8	TA	GG	GG	GA	GG	AA	TT	AG	TT	AA	AA	AG	GA	TT	GG	GG
m9	TT	GG	GA	AA	GG	AA	CC	GG	GG	TT	AT	AG	GG	AA	GG	AA
m10	AA	GG	GG	AA	AA	AA	TT	AA	TG	TT	AT	GG	GG	TT	GG	GG
m11	TA	GG	GG	GA	AA	GA	TC	AG	GG	TT	AT	GG	GA	TT	AA	GG
m12	TT	AG	AA	GG	GG	GG	TT	AA	TG	TT	TT	AG	GG	AA	AA	GG
m13	TA	GG	AA	NA	GG	AA	NA	GG	GG	AA	TT	AA	GG	AA	GG	GA
m14	TA	GG	AA	GA	AA	AA	TC	AG	NA	TT	AT	GG	AA	TT	GG	GG
m15	TA	AA	AA	GG	GG	GA	TC	GG	GG	AA	AT	AA	GG	TT	AA	GA
m16	TT	GG	AA	AA	GG	AA	CC	GG	GG	TT	AT	AG	GG	AA	GG	AA
m17	TT	GG	GG	GA	GG	GA	TC	GG	TG	AA	TT	GG	GG	TT	AA	GG
m18	AA	AA	GG	GA	GG	GG	TT	AA	TG	TT	TT	GG	AA	TT	GG	GA
m19	TA	AG	GG	AA	GG	AA	CC	GG	TG	AA	AA	GG	GG	AA	GG	AA
m20	TT	GG	AA	NA	AA	GG	CC	GG	GG	AT	TT	GG	GG	AA	AA	GG
m21	NA	GG	GG	AA	GG	AA	CC	GG	TG	TT	AT	GG	GG	AA	GG	GG
m22	TA	GG	AA	GG	NA	AA	TT	GG	TT	TT	TT	AA	GG	AA	AA	GA
m23	TT	GG	AA	AA	GG	AA	CC	GG	GG	TT	AT	AG	GG	AA	GG	AA
m24	TT	AA	AA	GG	GG	GG	TT	AA	TG	TT	TT	AG	GG	AA	AA	GG
m25	AA	GG	AA	AA	AA	GA	TT	AA	GG	TT	TT	GG	GG	TT	GG	GA
m26	TT	AA	GG	GG	GG	GG	TT	AA	TG	TT	TT	AG	GG	NA	AA	GG
m27	TT	AA	GG	GG	GG	GG	TT	AA	TG	TT	TT	GG	GG	AA	AA	GG
m28	AA	GG	GA	AA	AA	GA	TT	AA	GG	TT	TT	GG	GG	TT	GG	GA
m29	AA	GG	GG	AA	AA	GA	TC	GG	TT	TT	AA	AA	AA	TT	AA	GG
m30	TA	GG	GG	AA	GG	AA	TT	GG	GG	AT	AT	GG	GA	TT	GG	AA
m31	TA	GG	GG	GG	AA	AA	CC	AA	GG	AT	AT	GG	GG	AA	AA	GG
m32	TA	GG	GA	AA	GG	AA	TT	GG	GG	AT	AT	AA	AA	TT	GA	GA
m33	TT	AA	GG	AA	GG	GA	TC	GG	GG	AT	AT	AG	GG	AA	GG	AA
m34	TA	AG	AA	AA	GG	AA	CC	GG	GG	AA	TT	AG	GA	TT	GG	GA
m35	TA	GG	GA	GG	AA	GA	CC	GG	GG	AT	AT	AG	AA	TT	GA	GG
m36	TA	GG	AA	GA	GG	GG	TT	AA	GG	TT	TT	AG	AA	TT	AA	AA
m37	TA	AG	GG	AA	AA	AA	CC	AA	TG	TT	AT	GG	AA	TT	GG	GG
m38	AA	AA	NA	GG	GG	GG	CC	GG	TG	AA	AT	GG	GG	TT	GG	GA
m39	AA	AA	GA	GA	GG	GG	TT	AA	TG	TT	TT	GG	AA	TT	GG	GA
m40	TA	GG	GG	GG	AA	AA	TT	AA	GG	TT	TT	AA	AA	TT	GG	GA
m41	NA	AG	AA	GG	GG	GA	TC	GG	TT	AA	TT	AG	GG	TT	AA	GA
m42	TA	GG	AA	AA	AA	AA	CC	GG	TT	TT	AA	GG	GG	TT	GG	GA
m43	AA	AA	GA	GA	GG	GG	TT	AA	TG	TT	TT	GG	AA	TT	GG	GA
m44	TA	AA	AA	GG	GG	GA	TC	GG	GG	AA	TT	AA	AA	TT	GG	AA
m45	TA	GG	GG	GG	GG	GG	TT	AG	TT	AT	AT	GG	GG	TT	AA	GA
m46	TA	GG	AA	GG	AG	GA	TC	GG	GG	AA	AT	AG	GA	TT	GG	GA
m47	TA	GG	GG	GA	GG	GA	CC	AG	TT	TT	AA	AG	AA	TT	GA	GA
m48	AA	AG	GG	GA	GG	GG	TC	GG	TG	TT	AT	AG	AA	TT	AA	GA
m49	TT	AA	AA	AA	AG	GA	CC	GG	GG	AT	TT	GG	AA	TT	AA	GA
m50	AA	GG	GG	GA	AA	GG	TT	AA	GG	AA	AA	AA	GG	AA	GG	GA
m51	TA	GG	GG	AA	GG	AA	CC	AG	TG	TT	AT	GG	GG	AA	AA	GA
m52	AA	GG	AA	GG	GG	AA	CC	GG	TT	TT	AT	AA	GA	TT	GG	GA
m53	NA	AA	NA	GA	GG	GG	TT	AA	TG	TT	TT	GG	AA	TT	GG	GA
m54	AA	GG	NA	AA	AA	AA	CC	GG	TG	TT	AT	GG	GG	AA	AA	GA
m55	AA	AA	GA	GA	GG	GG	TT	AA	TG	TT	TT	GG	AA	TT	GG	GA
m56	TT	GG	AA	AA	GG	AA	CC	GG	TG	NA	AA	AA	GA	AA	GG	AA
m57	TA	AG	AA	AA	GG	AA	CC	GG	GG	AA	TT	AA	GA	TT	GG	AA
m58	TT	GG	GG	GA	GG	GA	TT	AA	GG	AT	AA	AA	AA	AA	AA	GG
m59	TA	AG	GA	AA	GG	GA	CC	GG	GG	AA	AT	AA	GG	AA	AA	AA
m60	AA	AA	AA	GG	GG	GA	TC	GG	TG	AA	AA	GG	GG	TT	GG	GG
m61	TA	AA	GG	GA	GG	GA	CC	AA	GG	AT	AA	AA	GA	TT	AA	GA
m62	AA	AA	GG	AA	AA	GG	NA	AA	GG	AA	AT	GG	GG	TT	GA	GA
m63	TA	AG	AA	AA	GG	GG	CC	GG	TT	AT	AT	AG	GG	AA	GG	AA
m64	TT	AA	GG	GG	GG	GA	TC	GG	GG	AA	TT	AA	GA	TT	GG	AA
m65	TT	GG	GG	GG	GG	AA	CC	GG	GG	AA	AA	GG	AA	AA	GA	GG
m66	TA	AA	AA	GG	GG	GA	TC	AA	TG	TT	TT	AG	GG	AA	GA	GA
m67	TT	AG	GA	AA	GG	AA	TC	GG	GG	AA	AA	AA	AA	TT	GG	GA
m68	TT	AA	AA	AA	GG	GA	TC	GG	GG	AT	AT	AG	GG	AA	GG	AA
m69	AA	GG	GG	AA	AA	GA	CC	GG	GG	TT	AA	GG	AA	TT	GG	GA
m70	TA	GG	AA	GA	AA	GA	TC	GG	TT	TT	AT	AG	AA	AA	AA	GA
m71	TA	GG	GG	GG	AA	GA	TT	AG	TT	TT	AA	GG	GG	NA	AA	GA
m72	TT	AA	AA	GG	GG	GG	TT	AA	TG	TT	TT	AG	GG	AA	AA	GG
m73	TA	GG	AA	AA	GG	AA	TC	GG	GG	AA	AT	AA	GA	TT	GG	GA
m74	TA	GG	AA	GA	GG	GA	TT	AG	TT	AT	AT	GG	GG	TT	GG	GG
m75	TT	AA	GG	GG	GG	AA	CC	GG	TG	AA	AT	GG	GG	AA	GA	GG
m76	TT	AA	GA	GG	GG	GG	TT	AA	TT	TT	TT	AG	GG	AA	AA	GG
m77	TA	AA	AA	GG	GG	AA	TC	GG	TG	AA	AT	GG	GG	AA	AA	GG
m78	TT	NA	GG	GG	GG	AA	CC	GG	TG	AT	TT	GG	GG	AA	GG	GG
m79	TT	GG	GG	GG	GG	AA	CC	GG	GG	AA	AA	GG	AA	AA	GA	GG
m80	TA	AA	AA	AA	AA	AA	TC	AA	GG	AA	AA	GG	GG	TT	GA	GG
m81	TT	GG	GG	GG	GG	GG	TC	AA	GG	AA	AT	AG	GA	TT	GG	AA
m82	TT	GG	GA	AA	GG	NA	TC	GG	GG	AA	AA	AA	GA	TT	GG	NA
m83	AA	AA	NA	GA	GG	AA	TT	GG	NA	TT	AT	GG	AA	TT	AA	GA
m84	TA	AG	AA	GG	GG	GG	TT	GG	TG	AA	TT	AG	GG	TT	NA	GA
m85	TA	GG	AA	GG	GG	GA	TT	GG	TT	NA	AT	GG	GA	AA	GA	GA
m86	TA	GG	GG	AA	GG	GA	TT	AG	GG	AA	AT	AA	AA	TT	AA	GG
m87	TT	AG	AA	GG	GG	AA	TT	GG	GG	AT	TT	AG	AA	TT	GG	GG
m88	TA	GG	GG	GG	AG	AA	CC	GG	TT	TT	AT	AG	GA	TT	GG	AA
m89	AA	GG	AA	GA	AA	AA	CC	GG	GG	TT	AT	GG	GG	TT	GG	AA
m90	AA	GG	AA	GA	AA	AA	CC	GG	TG	TT	AT	GG	GG	TT	GG	AA
m91	AA	GG	GG	GA	AG	AA	CC	GG	GG	TT	AT	AG	GA	TT	GG	AA
m92	AA	GG	AA	GG	GG	AA	CC	GG	TT	TT	AT	AA	GA	TT	GG	GA
m93	AA	GG	GG	AA	GG	AA	CC	GG	TT	TT	AA	GG	AA	TT	AA	GA
m94	TA	GG	GG	GG	AG	AA	CC	GG	TT	TT	AT	AG	GA	TT	GG	AA
m95	TT	AA	AA	GG	GG	GG	TT	AA	TG	TT	TT	AG	GG	AA	AA	GG
m96	TT	NA	GG	GA	AA	GA	CC	AA	NA	AA	AT	GG	AA	TT	AA	AA
m97	TT	AA	AA	GG	GG	GG	TT	AA	TG	TT	TT	GG	GG	AA	AA	GG
m98	TA	NA	GG	AA	GG	GA	CC	GG	GG	TT	TT	AA	AA	TT	GG	GG
m99	TA	AG	AA	AA	GG	GA	CC	GG	TT	AA	AT	GG	GG	NA	GG	NA
m100	TA	GG	AA	GG	AA	GA	TC	GG	GG	NA	AT	NA	AA	TT	GG	GA
m101	TT	AA	GG	GA	AA	GA	CC	AA	GG	AT	AT	AG	AA	TT	NA	AA
m102	TT	AA	AA	GG	GG	GG	TT	AA	TG	TT	TT	AG	GG	AA	AA	GG
m103	TT	NA	GG	GA	AA	GA	CC	NA	GG	AT	NA	AG	AA	TT	NA	AA
m104	TT	AA	GG	GA	AA	GA	CC	AA	GG	AT	AT	AG	AA	TT	GA	AA
m105	TT	AA	AA	GG	GG	GG	TC	AG	TG	TT	TT	GG	GG	TT	GA	GG
m106	TT	AA	AA	GG	GG	AA	CC	GG	TG	AA	AT	GG	GG	AA	NA	GG
m107	TA	AG	GG	GG	GG	GA	CC	AA	GG	AT	AT	GG	GG	AA	AA	AA
m108	TA	AA	AA	AA	AA	GG	CC	AG	TG	AA	AA	GG	GA	AA	GG	GG
m109	AA	GG	GG	AA	AA	GA	CC	GG	TT	TT	AA	AA	AA	TT	AA	GG
m110	TA	GG	GG	GG	AA	GA	TT	AA	TT	TT	AA	GG	GG	AA	GG	GA
m111	AA	GG	GA	GG	AA	GA	CC	AA	TT	AT	AT	GG	AA	TT	GG	GA
m112	TA	GG	AA	GG	AG	GA	CC	AA	GG	TT	AA	AG	AA	TT	GG	GG
m113	AA	GG	GA	GG	AA	GA	CC	AA	TT	TT	AT	GG	GA	TT	GG	GA
m114	TT	GG	AA	AA	AA	GA	CC	GG	TT	TT	AA	AA	AA	AA	GA	AA
m115	AA	AA	AA	GG	GG	AA	CC	AA	TG	TT	TT	GG	GG	AA	GG	GA
m116	TT	AA	GG	GA	AA	GA	CC	AA	GG	AT	AT	AA	AA	TT	GA	AA
m117	TA	AA	AA	GA	GG	GA	TC	AG	TG	TT	TT	NA	AA	TT	GA	GG
m118	TT	AA	GG	GA	AA	GA	CC	NA	GG	AT	AT	NA	AA	TT	NA	AA
m119	TT	AA	GG	GA	AA	GA	NA	AA	GG	TT	AT	GG	AA	TT	AA	AA
m120	TT	AA	GG	GA	AA	NA	CC	AA	GG	AT	AT	NA	AA	TT	AA	AA
m121	AA	AA	GG	AA	AA	GG	TC	AA	GG	AT	AT	GG	GG	TT	AA	GA
m122	TA	GG	AA	AA	GG	GA	CC	AG	TG	AA	AT	AA	GA	AA	GA	AA
m123	TA	AA	AA	AA	GG	AA	CC	GG	TG	TT	AA	GG	GG	AA	AA	AA
m124	AA	AA	GA	GA	GG	GG	TT	AA	TG	TT	TT	GG	AA	TT	GG	GA
m125	TA	GG	AA	AA	GG	AA	CC	GG	TT	AA	AT	AG	GG	AA	GG	AA
m126	TA	AA	AA	AA	GG	AA	CC	GG	TG	TT	AA	GG	GG	AA	AA	AA
m127	TA	GG	AA	AA	AA	GA	CC	AA	TG	AT	TT	AG	GG	AA	AA	GA
m128	TT	AA	GG	GA	AA	GA	CC	AA	GG	TT	AT	AG	AA	TT	AA	AA
m129	TA	GG	GG	GG	AA	AA	CC	GG	TT	TT	AT	AG	GA	TT	GG	AA
m130	TT	AA	AA	GG	GG	GG	TT	AA	TG	TT	TT	AG	GG	AA	AA	GG
m131	TT	AA	AA	GG	GG	GG	TT	AA	TG	AA	TT	AG	GG	AA	AA	GG
m132	AA	AA	AA	GG	GG	GA	TC	GG	TG	TT	AA	AA	GG	TT	GG	GG
m133	AA	GG	AA	AA	GG	GA	TT	GG	TG	AT	AT	GG	AA	TT	GG	GG
m134	TT	AG	GG	AA	GG	GA	TC	GG	GG	AT	AT	AG	GG	TT	GG	AA
m135	TT	AA	GG	GA	AA	GA	CC	AA	GG	AA	AT	AG	GA	TT	AA	AA
m136	TT	AG	AA	AA	GG	AA	CC	GG	TT	TT	AA	AG	GG	AA	AA	AA
m137	AA	GG	GG	GA	GG	AA	CC	GG	TT	TT	TT	AA	AA	TT	GG	AA
m138	AA	GG	AA	GG	GG	AA	CC	GG	TT	AT	AT	AA	AA	TT	GG	GA
m139	TT	AG	GG	GA	AG	GA	CC	AG	GG	AT	AT	NA	AA	TT	GG	AA
m141	AA	AA	AA	GA	AG	GG	CC	AA	GG	TT	AT	AG	AA	TT	GG	GA
m142	AA	GG	GG	GG	AA	AA	TC	GG	NA	TT	TT	AG	AA	TT	GG	GA
m143	TA	GG	AA	AA	GG	AA	TC	GG	TT	TT	AT	GG	GG	TT	GG	AA
m144	AA	GG	GG	GA	AA	AA	TC	AG	TT	TT	AA	GG	GA	TT	GA	GA
m145	TA	GG	GG	NA	NA	NA	CC	GG	TT	AA	AT	AG	GA	TT	AA	GA

NA is the unrecognized sample in this study

## Abbreviations

SNP: Single nucleotide polymorphism
PARMS: Penta-primer Amplification Refractory Mutation System
PIC: Polymorphic information content
MAF: Minor allele frequency
PCA: Principal component analysis
PCR: Polymerase Chain Reaction
RAPD: Random Amplified Polymorphic DNA
ISSR: Inter-simple sequence repeat
SSR: Simple sequence repeats
CAPS: Cleaved Amplified Polymorphism Sequences
SSCP: Single Strand Conformation Polymorphism
HRM: High-Resolution Melting
KASP: Kompetitive Allele specific PCR
SRAP: Sequence-Related Amplified Polymorphism
CDDP: Conserved DNA-derived polymorphism
CEAP: Computer Animation Production System
