# Supplementary material for: Development and application of whole genome SNP markers in mango
Source: BMC Genomics. 2026 Jul 18;27:886. doi: 10.1186/s12864-026-13182-z (PMC13700291; doi:10.1186/s12864-026-13182-z)
Supplement: Supplementary file 1 [file 12864_2026_13182_MOESM1_ESM.pdf]

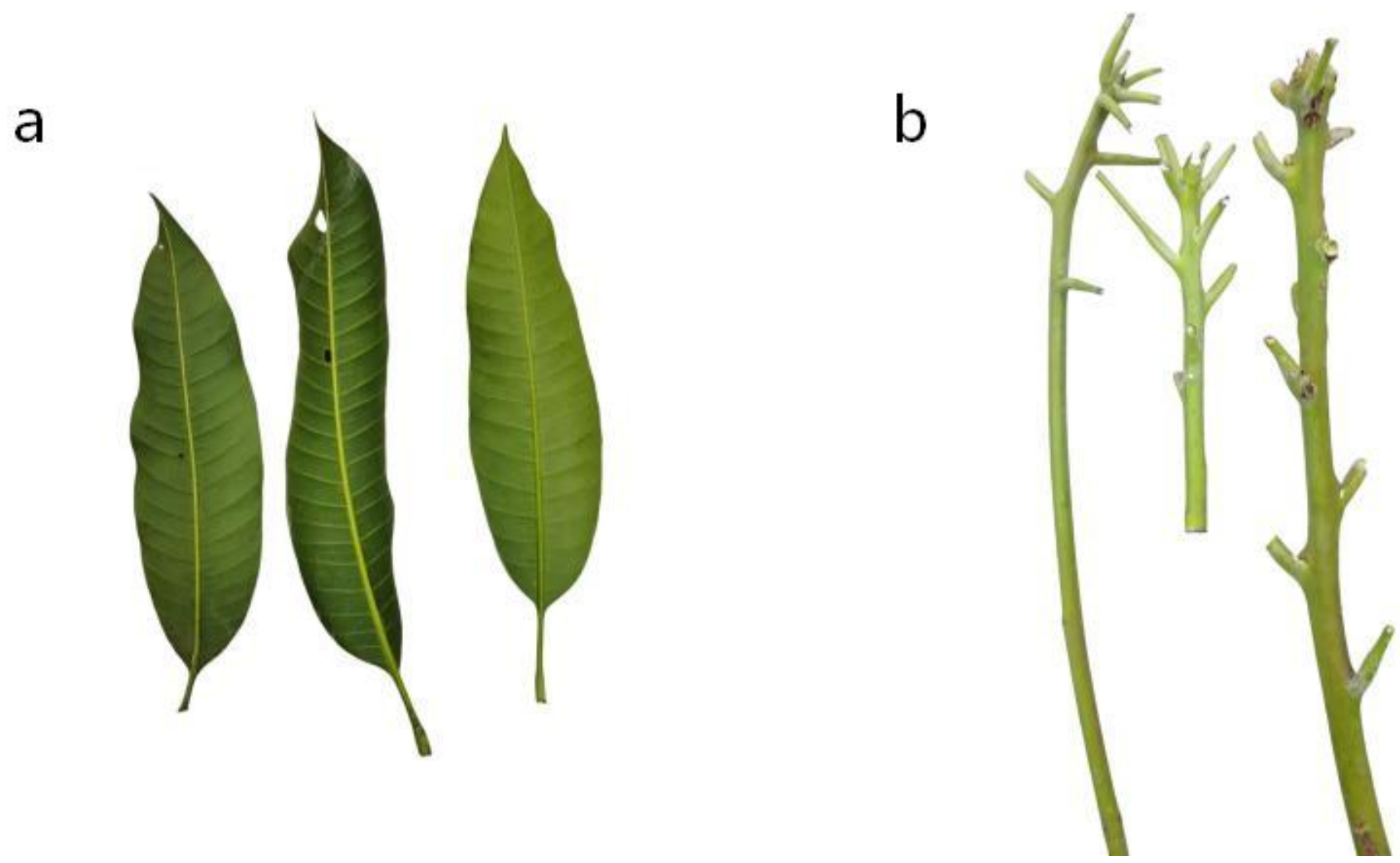

Supplementary material Fig.1 Morphological Characteristics of M95 (Small Philippine Mango), M24(Guire No.10) , and M102 (Guangxi 8) :

(a) the leaf traits of M95 (Small Philippine Mango), M24 (Guire No.10) and M102 (Guangxi 8) from left to right ; (b) The stem and branch traits of M95 (Small Philippine Mango), M24 (Guire No.10) and M102 (Guangxi 8) were from left to right.
